# Assessing Patient-Reported Outcomes in Routine Cancer Clinical Care Using Electronic Administration and Telehealth Technologies: Realist Synthesis of Potential Mechanisms for Improving Health Outcomes

**DOI:** 10.2196/48483

**Published:** 2023-11-28

**Authors:** Ramkumar Govindaraj, Meera Agar, David Currow, Tim Luckett

**Affiliations:** 1 Department of Radiation Oncology Royal Adelaide Hospital Adelaide Australia; 2 Faculty of Health and Medical Sciences University of Adelaide Adelaide Australia; 3 IMPACCT - Improving Palliative, Aged and Chronic Care through Clinical Research and Translation Faculty of Health University of Technology Sydney Sydney Australia; 4 Faculty of Science Medicine and Health University of Wollongong Wollongong Australia

**Keywords:** patient-reported outcome measure, PROM, PROMs, realist synthesis, oncology, eHealth, patient reported, outcome measure, outcome measures, realist, literature review, narrative review, search strategy, review methods, review methodology, electronic patient-reported outcome measure, ePROM, cancer, oncology, self-reporting, mobile phone

## Abstract

**Background:**

The routine measurement of patient-reported outcomes in cancer clinical care using electronic patient-reported outcome measures (ePROMs) is gaining momentum worldwide. However, a deep understanding of the mechanisms underpinning ePROM interventions that could inform their optimal design to improve health outcomes is needed.

**Objective:**

This study aims to identify the implicit mechanisms that underpin the effectiveness of ePROM interventions and develop program theories about how and when ePROM interventions improve health outcomes.

**Methods:**

A realist synthesis of the literature about ePROM interventions in cancer clinical care was performed. A conceptual framework of ePROM interventions was constructed to define the scope of the review and frame the initial program theories. Literature searches of Ovid MEDLINE, Ovid Embase, Scopus, and CINAHL, supplemented by citation tracking, were performed to identify relevant literature to develop, refine, and test program theories. Quality appraisal of relevant studies was performed using the Mixed Methods Appraisal Tool.

**Results:**

Overall, 61 studies were included in the realist synthesis: 15 (25%) mixed methods studies, 9 (15%) qualitative studies, 13 (21%) descriptive studies, 21 (34%) randomized controlled trials, and 3 (5%) quasi-experimental studies. In total, 3 initial program theories were developed regarding the salient components of ePROM interventions—remote self-reporting, real-time feedback to clinicians, and clinician-patient telecommunication. The refined theories posit that remote self-reporting enables patients to recognize and report symptoms accurately and empowers them to communicate these to clinicians, real-time feedback prompts clinicians to manage symptoms proactively, and clinician-patient telephone interactions and e-interactions between clinic encounters improve symptom management by reshaping how clinicians and patients communicate. However, the intervention may not achieve the intended benefit if ePROMs become a reminder to patients of their illness and are not meaningful to them and when real-time feedback to clinicians lacks relevance and increases the workload.

**Conclusions:**

The key to improving health outcomes through ePROM interventions is enabling better symptom reporting and communication through remote symptom self-reporting, promoting proactive management of symptoms through real-time clinician feedback, and facilitating clinician-patient interactions. Patient engagement with self-reporting and clinician engagement in responding to feedback are vital and may reinforce each other in improving outcomes. Effective ePROM interventions might fundamentally alter how clinicians and patients interact between clinic encounters.

## Introduction

Interest in using patient-reported outcome measures (PROMs) in routine cancer care has steadily increased. Until recently, systematically collecting patient-reported outcomes in routine cancer care to improve health outcomes, such as health-related quality of life (HRQOL), has not been supported by evidence. However, the impact on other measures, such as patient satisfaction and clinician-patient communication, has been consistently demonstrated [1-3]. Hence, the recent publication of studies [4-7] showing the positive impact of PROM intervention on health outcomes is pivotal because it provides the much-awaited proof of principle that the routine use of PROMs in cancer clinical care can improve health outcomes, including HRQOL and even survival.

There are important differences between the recent studies that have shown a positive impact on health outcomes and many other studies performed over the past decade. The differentiating feature of the recent successful studies has been the use of modern electronic PROM (ePROM) interventions, which possess additional features that enable remote symptom reporting, real-time feedback, and alerts to clinicians. Although it does not necessarily follow that electronic administration is the only feature of the new interventions that might have led to positive effects on outcomes, recent studies suggest that this mode of administration may be contributory [7-9]. Moreover, electronic administration of PROMs is rapidly becoming the norm, and several ePROM programs have already been rolled out in many clinical settings [8,10-12].

Modern ePROM interventions are not merely electronic versions of paper-based PROMs but function more as eHealth interventions. The World Health Organization defines eHealth as “the cost‐effective and secure use of information and communications technology in support of health and health-related field” [13]. eHealth interventions have been conceptualized in many ways; however, the key elements include technology to track or monitor health information, enabling clinician-patient communication, and analysis and management of health-related data to enable health [14]. Modern ePROM interventions use web-based or mobile platforms or both and can collect PROM data in real time, which could be used to track patients’ symptoms outside the clinic setting. They also enable immediate electronic feedback of PROM reports to clinicians, allowing real-time monitoring of patients’ symptoms [10]. With these features, the ePROM interventions function more or less similarly to eHealth interventions. Furthermore, many ePROM interventions also provide tailored feedback to patients for self-monitoring and real-time alerts to clinicians to enable early identification of changes in patients’ health status. However, how and which components of ePROM interventions contribute to improvement in health outcomes is poorly understood [15,16], and as a digital intervention, there is currently insufficient theoretical grounding to inform their future practice and development [17].

To unravel complex interventions and understand the mechanisms behind what drives the realization of outcomes, realist synthesis [18,19], a theory-driven approach, has been used to synthesize the literature about PROM interventions. However, the existing realist syntheses [20-22] that explore the theoretical underpinnings of the general PROM interventions do not focus on modern ePROM interventions and broadly assess PROM interventions across many clinical settings. As the outcomes of any intervention are determined by the context in which they are undertaken, focusing on the cancer clinical setting may reduce “noise” from disease-related issues that have interfered with previous attempts to identify key pathways. This study aimed to develop and test program theories about how ePROM interventions would improve health outcomes, what attributes of the ePROM interventions are important, and in what context they are likely to produce the desired outcomes in routine cancer care.

## Methods

### Overview

The overarching research question for this study was “How do ePROM interventions improve health outcomes in routine cancer care?” The methodology was based on the tenets of realist synthesis outlined by Pawson et al [18] and guidelines provided by the Realist and Meta-Narrative Evidence Syntheses–Evolving Standards training material [23]. The Realist and Meta-Narrative Evidence Syntheses–Evolving Standards guidelines was followed to report the methods and results [24]. The review’s objectives were to formulate and test program theories about how, under what circumstances, and for whom ePROM interventions improve health outcomes through the following:

1. Determining the attributes (resources) that are important to improve health outcomes in an ePROM intervention
2. Identifying important contextual factors that determine when ePROM interventions are likely to be effective
3. Identifying the mechanisms underpinning the effectiveness of ePROM interventions in improving health outcomes

There were 3 broad stages in this realist synthesis. The first stage involved defining the scope of the review and formulating initial program theories (IPTs), which was accomplished by reviewing the current evidence base gathered through a scoping, informal literature search. IPTs are assumptions or propositions describing how ePROM interventions worked in practice and produced the desired outcomes. In the second stage, IPTs were developed into a series of explanatory statements—context-mechanism-outcome (CMO) configurations—informed by empirical literature gathered through formal literature searches. In a realist synthesis, CMO configurations represent hypotheses explaining how a particular context or contexts activate mechanism or mechanisms, which are often implicit, to generate outcomes; all plausible CMO configurations are sought that explain not only how outcomes are produced but also why they may not be produced. The third stage involved testing and refining the CMO hypotheses using empirical literature to confirm, reject, or modify them. The second and third stages were conducted nonlinearly, where building and testing CMO configurations involved an iterative process moving back and forth between the initial and refined CMO configurations as the evidence synthesis evolved.

### Stage 1: Scope of the Review and Theory Mining

The review’s focus was refined through research team meetings among the authors. A conceptual framework of how ePROM interventions have been used in practice was first constructed to define the scope, thereby identifying the key theoretical notions behind the working of ePROM interventions. For this purpose, an informal literature search was performed, supplemented by citation tracking of prominent papers in this field. Previously published realist reviews [20-22,25] were also consulted. The key components of ePROM interventions and concepts were drawn from the literature to construct the ePROM framework.

Using the conceptual framework, we identified distinct features of an ePROM intervention that enable it to function as an eHealth intervention. The premise was that identifying these elements would enable theory mining to focus on the aspects of ePROM interventions that differentiate them from traditional non-ePROM and ePROM interventions that do not function as eHealth interventions. Furthermore, the developed program theories would then explain how these elements of ePROMs produce the desired outcomes. To distinguish ePROM interventions that are truly implemented as eHealth interventions and not merely electronically administered PROMs, we defined ePROM intervention as an eHealth intervention in terms of 2 essential elements: remote ePROM completion and electronic feedback to the clinician. The ePROM systems may have other functionalities such as tailored patient feedback but were not considered necessary. We also focused on health outcomes as the outcomes of interest but included health outcome–related process outcomes.

The conceptual framework was then used to develop broad theoretical concepts (theory areas) and rough IPTs. The IPTs were conceptualized through deliberations among the authors, informed by the evidence from the scoping, informal literature search and the constructed conceptual framework, and it involved using some amount of creative thinking and hunches about how ePROMs worked in practice to achieve improvement in health outcomes.

### Stages 2 and 3

#### Literature Search

In the second stage, a formal systematic literature search was performed to identify literature, develop the IPTs, and refine and test them. The search scope was limited to studies using ePROMs in the cancer clinical care setting. A search strategy was developed for the Ovid MEDLINE database before translating it for other databases—Ovid Embase, Scopus, and CINAHL (Multimedia Appendix 1). The key search terms used to develop the search strategy were “patient-reported outcomes,” “electronic patient-reported outcomes,” “self-report,” “neoplasm,” “telemedicine,” “eHealth,” and “mHealth.” Gray literature was also searched using Google Search. Other search methods were forward and backward citation tracking (using Scopus) of important publications and a “snowballing” bibliography search of these papers. No year limits were applied to the search. Given the focus of the review on ePROMs in routine adult cancer care, studies using PROMs as an outcome measure to evaluate an intervention and studies in children or focusing only on the psychometric properties of PROMs were excluded. Studies not published in English were also excluded owing to lack of resources for translation. All types of studies—qualitative, quantitative, and mixed methods—were included.

An additional, focused literature search was also conducted to identify studies of remote symptom monitoring because ePROMs were used predominantly as a symptom monitoring tool during active cancer treatment. The focused search also aimed to identify empirical studies that could be used to link theories to the realization of health outcomes. The keywords used were “patient-reported outcomes,” “electronic patient-reported outcomes,” “remote monitoring,” “real-time monitoring,” “symptom monitoring,” “neoplasm,” “telemedicine,” “eHealth,” and “clinical outcome.” Only the Ovid MEDLINE database was searched for this iteration of the literature search. The inclusion criteria for the focused search were studies (experimental, quasi-experimental, or analytical observation studies) evaluating ePROM intervention specifically as a symptom monitoring tool and reporting on health outcomes: adverse events, symptom severity, HRQOL, survival or progression-free survival, or health outcome–related process outcomes such as unplanned health care use (hospital or emergency department admission) and treatment adherence. The inclusion and exclusion criteria are provided in Multimedia Appendix 1.

#### Data Extraction and Quality Appraisal of Studies

The results of the first formal literature search were filtered using broad selection criteria framed as questions:

1. “Does the article address any aspect of ePROM intervention?”
2. “Is the article relevant to the cancer clinical care setting?”

This process judged the “relevance” of the paper, as described by Pawson et al [18]. “Relevance” and “rigour” are the 2 suggested criteria recommended by Pawson et al [18] to appraise literature in a realist synthesis. Relevance was judged in terms of whether a paper is relevant to the topic being studied and the richness of the information a paper can contribute toward building program theories. We further screened the literature for relevance to the topic of interest using the inclusion criteria (Multimedia Appendix 1) and operationalized richness by categorizing papers into high and low relevance:

High relevance—papers with relevant empirical data explicitly describing theories and concepts or enabling the inference of underpinning theories

Low relevance—papers with no relevant empirical data, scarce descriptions of concepts that can be used to build or refine theories, or limited descriptions of interest that were already known from other papers of high relevance

The second focused search was also screened similarly using the inclusion criteria (Multimedia Appendix 1) for relevance to the topic. In a realist synthesis, relevance often evolves as theories are developed, and the appraisal of richness is adapted to the changes in what is relevant to the theories being developed. Consistent with this feature of realist synthesis, the assessment of richness of the content of the literature was adapted for the second focused search—as the second iteration of the formal literature search aimed to identify empirical studies of symptom monitoring that enabled conceptualizing how health outcomes were achieved, we categorized papers as highly relevant if they provided relevant empirical data or conceptualized the process and described how health outcomes were generated, contributing to theory testing and refining. Only papers categories as highly relevant were included from both searches.

Rigor was assessed at the data source and theory levels [26]. To assess the rigor of the included studies, a validated assessment tool, the Mixed Methods Appraisal Tool version 2018 [27], was used, which assesses the trustworthiness at the data source level. We appraised the methodological quality of all the studies used in the synthesis that were critical for theory development and testing, where the methodological quality of the whole study was important for the validity of the information extracted from the study. We deemed it unnecessary to provide the quality appraisal of the studies from which the contributing piece of information for theory development was drawn from a small section, the validity of which did not depend on the methodological quality of the empirical data or the whole study. No studies were excluded based on quality assessment. Finally, we evaluated the rigor at the theory level to indicate the overall coherence of the theory, which suggests how much each theory was supported by the literature [26].

Data extraction was performed using a bespoke data extraction sheet formulated using the information from the conceptual framework (Multimedia Appendix 2). It contained separate sections for each theory area identified from the conceptual framework, and the data contributing to each theory area were placed in the respective sections. Any unintended and new theory areas identified in the papers were also extracted. A description of the ePROM study design, components of the ePROM intervention, study methodology, and study results were also extracted. The data were coded using NVivo (Lumivero) software.

#### Synthesis

The studies’ extracted data were first identified as related to the resource, context, outcomes, or information that would aid in hypothesizing the underlying mechanisms. We then analyzed the data and used the realist principles of generative causation to conceptualize the implicit mechanism that could explain how outcomes of interest were produced in the presence of a particular context [18,24]. We identified recurring context, mechanism, and outcome patterns across studies and formulated composite CMO configurations, which were placed under relevant IPTs. We used descriptive, qualitative, and mixed methods studies to derive the CMO configurations. To test the IPTs, we first identified supportive evidence that indicates the operation of the causal mechanisms that underpin the IPTs and then identified evidence to substantiate that it improved health outcomes. As far as possible, we sought evidence from comparative effectiveness studies to confirm or refute that the putative mechanisms linked to an aspect of the ePROM intervention led to improved health outcomes. Where the supportive evidence was derived from separate studies, we scrutinized the evidence in stages: identified studies that support the causal mechanisms first and then those that provide evidence for improvement in health outcomes. We revised and reframed the IPTs through an iterative theory testing process based on the available evidence.

The protocol for the review was registered in PROSPERO (CRD42020221238). The following protocol deviation occurred during the review. As there was a substantial number of mixed methods studies among the included studies, instead of an initial plan to use the Joanna Briggs Institute critical appraisal tools, Mixed Methods Appraisal Tool was used to appraise the quality of the studies.

### Ethics Approval

Ethics approval was not obtained because this review used publicly available literature.

## Results

### Conceptual Framework and IPTs

**Figure 1:**
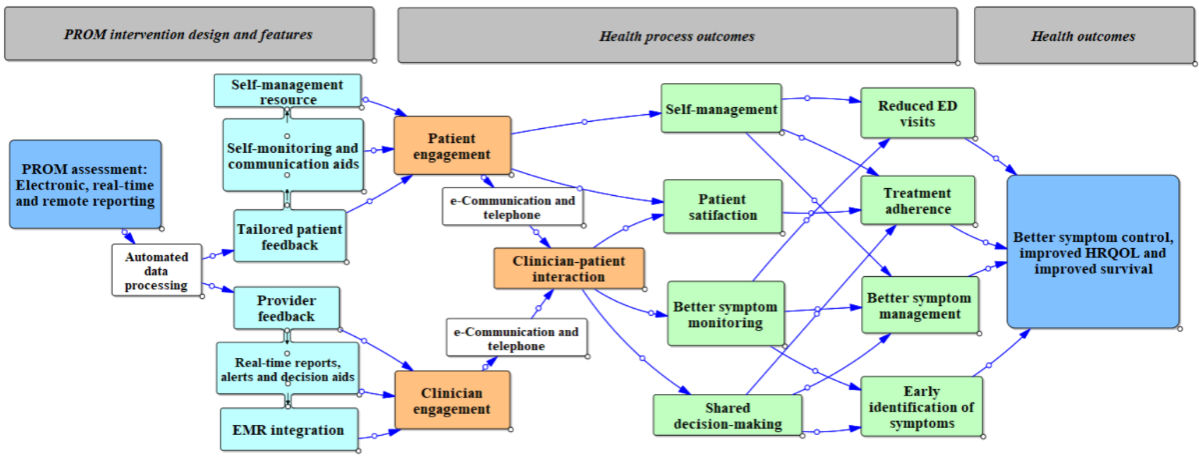
Conceptual framework of electronic patient-reported outcome measure (PROM) intervention. ED: emergency department; EMR: electronic medical record; HRQOL: health-related quality of life.

The conceptual framework of ePROM interventions is shown in Figure 1, illustrating the various components used in the architecture of ePROM interventions and the potential impact on health processes and outcomes. Overall, 14 papers informed the construction of the framework: 3 (21%) editorial comments, 1 (7%) educational article, 1 (7%) symposium presentation, 1 (7%) descriptive study, and 8 (57%) reviews [10-12,15-17,28-35].

We identified 3 main “theory areas” representing broad principles of how ePROM interventions could improve outcomes: patient engagement, clinician engagement, and clinician-patient interaction (Textbox 1). The conceptual framework (Figure 1) demonstrates how these 3 theory areas are linked to the components (resources) of ePROM interventions and the outcomes. Overall, 3 rough IPTs were conceptualized within the theory areas (Textbox 1).

**Theory areas and initial program theories (IPTs).**

**Patient engagement (IPT1)**

- Remote self-reporting enables patients to recognize and report symptoms accurately through better awareness and insights about their illness. It empowers patients to communicate symptoms to their health care team, enabling early identification.

**Clinician engagement (IPT2)**

- Real-time feedback provides clinicians with a better picture of patients’ symptom experience, which, when combined with alerts and red flags, enables early identification of symptoms and promotes proactive management.

**Clinician-patient interaction (IPT3)**

- Clinician-initiated telephone interactions and e-interactions between clinic visits in response to patients’ self-reports facilitate timely patient-centered interactions, enabling prompt symptom management.

### Literature Search and Characteristics of the Included Studies

**Figure 2:**
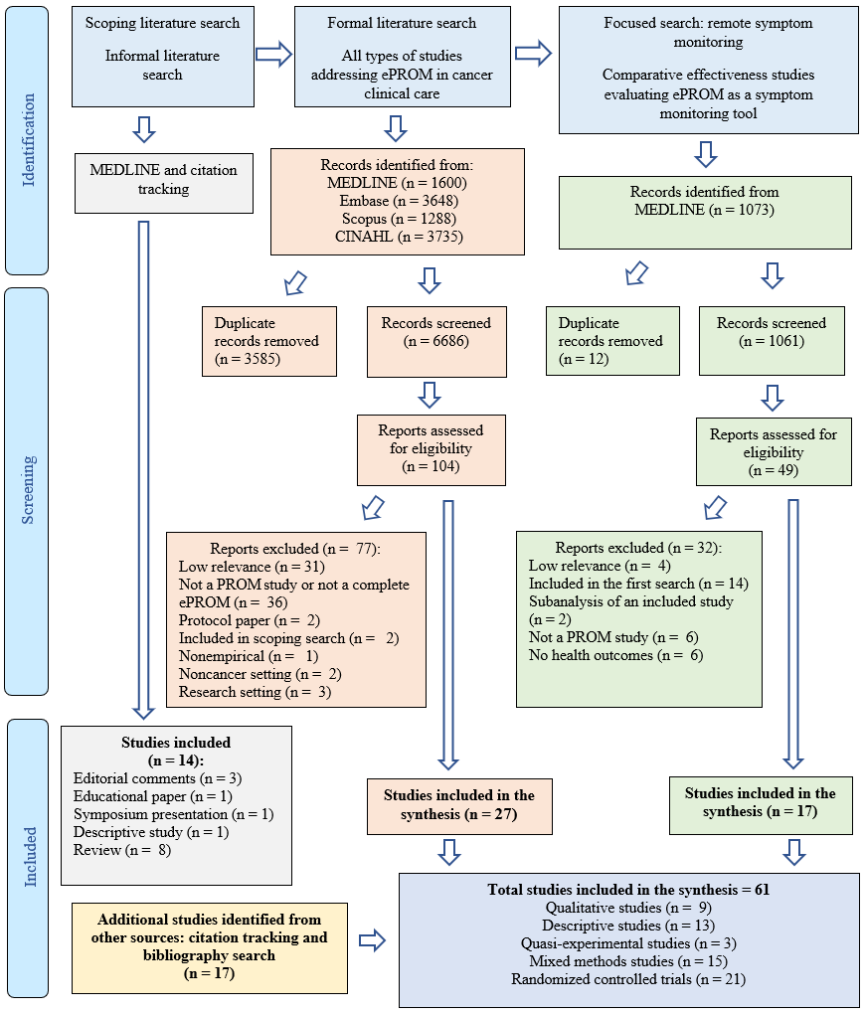
PRISMA (Preferred Reporting Items for Systematic Reviews and Meta-Analyses) flow diagram illustrating the literature searches. ePROM: electronic patient-reported outcome measure; PROM: patient-reported outcome measure.

The formal literature search produced 6686 citations (Figure 2). After screening the citations, 4.89% (327/6686) of the papers were shortlisted for title and abstract screening. The title and abstract screening yielded 1.56% (104/6686) of relevant papers that were subjected to full-text examination, and 0.40% (27/6686) were found to be relevant and included in the synthesis. Overall, 17 papers were identified through citation tracking and other searches. The focused MEDLINE search for literature about remote symptom monitoring identified 17 empirical studies. In total, 25% (15/61) of mixed methods studies, 15% (9/61) of qualitative studies, 21% (13/61) of descriptive studies, 34% (21/61) of randomized controlled trials (RCTs), and 5% (3/61) of quasi-experimental studies were used in the synthesis. The characteristics of the included studies are provided in Multimedia Appendix 3 [4-9,36-91].

The methodological quality of pertinent studies that provided crucial evidence to test theories is discussed, where the respective studies appear in the Results section. Quality appraisal of individual studies is provided in Multimedia Appendix 4 [4,6-9,42-91]. Overall, 5% (3/61) of qualitative studies [36-38], 2% (1/61) of descriptive study [39], and 3% (2/61) of mixed methods studies [40,41] did not undergo quality appraisal because the contribution from these studies was minimal and was not affected by the overall quality of the study.

### Synthesis

#### Overview

We identified 3 CMO configurations (Table 1) about how remote self-reporting of symptoms with feedback to the clinician could improve health outcomes and 1 rival CMO configuration about why it may not; 2 CMO configurations under IPT 2 describing how real-time monitoring may improve health outcomes; and 1 CMO configuration under IPT 3 about how ePROM-enabled clinician-patient communication could improve health outcomes.

**Table 1:** Context-mechanism-outcome (CMO) configurations.

IPT^a^ number and CMO configuration		Context	Mechanism	Outcome	Literature source
**IPT 1**					
	1.1—Remote symptom self-reporting supports better symptom reporting	Patients are more likely to experience symptoms at home between clinic visits and after discharge from the hospital and may struggle to recall these symptoms at clinic encounters.	Remote symptom reporting enables patients to observe, reflect, and recognize their symptoms better because they are in their familiar environment, without the distractions and time constraints of a clinic environment. By allowing patients to report symptoms at any time, they can accurately report symptoms, thus eliminating recall issues.	Better symptom identification and accuracy of symptom reporting	Snyder et al [42], Brochmann et al [43], Graetz et al [44,46], Falchook et al [45], Bae et al [47], and Wintner et al [48]
	1.2—By self-reporting more frequently, patients gain better awareness of symptoms	Patients’ illness experience is dynamic; is shaped by treatment, environmental, and social contexts; and fluctuates over time. Symptom fluctuations between clinic visits are often unrecognized and less likely to be incorporated into clinic consultations.	Frequent self-reporting of symptoms with a graphical display of symptom scores and time trends enables patients to understand their health status better through self-reflection and by recognizing patterns in their symptom experience.	Patients develop better insights into their health status, prioritize their problems, and better remember symptoms during consultation with clinicians	Crafoord et al [49], Tolstrup et al [50], Whitehead et al [51], Gustavell et al [52], Dawes et al [53], Basch et al [54], Snyder et al [42,55], Andikyan et al [56], Lee et al [57], Richards et al [58], Biran et al [59], Warrington et al [60], and Wu et al [61]
	1.3—Remote self-reporting as a communication tool	Patients feel isolated between clinic visits as they lack contact with their clinicians. However, they are hesitant to contact clinicians owing to uncertainty about whether their symptoms warrant a call and are worried about bothering the clinicians.	Patients’ awareness that the clinicians monitor their self-reports makes them feel reassured and connected; e-communication via self-reports encourages patients to communicate their concerns to their clinicians without hesitancy or guilt about disturbing clinicians by calling them, thus empowering patients to communicate. Automated, tailored patient feedback about when to contact clinicians may also give patients the legitimacy to contact clinicians.	Improves patient-centered communication and enables early symptom management	Girgis et al [62], Snyder et al [42,55], Tolstrup et al [50,68], Wu et al [61], Cox et al [36], Gustavell et al [52], Lee et al [57], Denis et al [39], Falchook et al [45], Wintner et al [48], Basch et al [7,54,63], Sundberg et al [65], Zivanovic et al [41], McCann et al [37], Moradian et al [40], Maguire et al [66], Richards et al [58], Taylor et al [38], Duman-Lubberding et al [67], Dawes et al [53], and Crafoord et al [49]
	1.4—ePROMs^b^ may become a burden and a reminder of illness	Patients must repeatedly confront their illness and symptoms when they self-report using ePROMs.	Patients might view ePROM self-reporting as a negative reminder of their illness if their symptoms are chronic and not amenable to effective treatment; they might view it as a burden if ePROMs do not resonate with their situation and illness.	Disengagement of patients from self-reporting	Crafoord et al [49], Tolstrup et al [50], Maguire et al [66], Egbring et al [69], Brochmann et al [43], Snyder et al [55], Duman-Lubberding et al [67], Richards et al [58], Hansen et al [70], McCann et al [37], Biran et al [59], Basch et al [63], and Lee et al [57]
**IPT 2**					
	2.1—Promoting proactive symptom management by clinicians through real-time feedback of symptom reports	Patients are most vulnerable between clinic encounters because clinicians are unaware of their problems during this period.	Real-time feedback to clinicians of remotely completed symptom reports enables clinicians to better understand patients’ illness experiences, which are otherwise unavailable to clinicians. Lack of contact with patients between clinic encounters may prime clinicians to react to feedback reports received during this period, and when symptom reports trigger alerts, clinicians are more likely to identify the information as critical, prompting them to act proactively.	Timely detection and prompt management of symptoms	Duman-Lubberding et [67], Girgis et al [62], Snyder et al [55], Tolstrup et al [50], Maguire et al [66,79,80], Cox et al [36], Coolbrandt et al [76], Mooney et al [81], Whitehead et al [51], and Sundberg et al [65]
	2.2—EMR^c^ integration of ePROMs and ease of use encourages clinicians to use the feedback	Clinicians often perceive including feedback reports in clinical care as time consuming and will only review them if they are readily usable.	Providing feedback reports, highlighting meaningful and key changes with decision aids, encourages clinicians to use the feedback reports. Integrating feedback reports into EMR and workflow encourages clinicians to use ePROM feedback because ePROM reports will be available alongside patients’ clinical details and laboratory reports, which provides context to the ePROM reports. It simulates their routine clinical work and may motivate clinicians to view it as integral to patients’ care.	Early identification and prompt management of symptoms	Basch et al [63,75], Maguire et al [66,80], Tolstrup et al [50], Simon et al [82], Biran et al [59], Wu et al [61], Snyder et al [42,55], Taylor et al [38], and Bae et al [47]
**IPT 3**					
	3.1—ePROM-enabled telecommunication reshapes clinician-patient interactions	When patients are provided with ePROMs to self-report remotely, they are keen to use it to communicate their concerns to their clinicians. However, they might abandon self-reporting if it does not foster interaction with their clinicians.	When patients receive prompt feedback from their clinicians (clinician-initiated interactions) for their self-reports between clinic visits, they feel acknowledged and perceive clinicians as being sensitive to their concerns and willing to respond to their needs, reinforcing their participation in self-reporting.	Patient-centered interaction between clinic encounters focused on patients’ issues expressed through self-reports, enabling early detection and better management of symptoms before clinic encounters	Wu et al [61], Hansen et al [70], Lee et al [57], Bae et al [47], Snyder et al [55], Cleeland et al [83], Biran et al [59], Tolstrup et al [50], Maguire et al [79], Dawes et al [53], and Basch et al [75]

^a^IPT: initial program theory.

^b^ePROM: electronic patient-reported outcome measure.

^c^EMR: electronic medical record.

#### IPT 1

##### CMO 1.1: Remote Symptom Self-Reporting Supports Better Symptom Reporting

A possible mechanism could be that remote symptom reporting leverages patients’ better awareness of symptoms within their home environment because patients are not as anxious or distracted as in a clinic environment. The home environment and available time may also provide calmness for patients to self-reflect and report their symptoms [42,43]. Quotes from patient interviews in the studies by Snyder et al [42] and Graetz et al [44] imply this mechanism:

A lot of these questions...I could sit at home and answer, and think about it a little.... So I think this is very good. [42]

I think it was very helpful and, like I say, letting me think about how I did feel that day and answer the questions as truthfully as possible for the feedback to get back to the proper people in case something was wrong. [44]

Moreover, patients can report symptoms as they occur, enabling them to accurately report them without being affected by recall issues [45]. Graetz et al [46] reported a patient’s quote that aligns with our assumption:

I can go in and note my symptoms before I went to see the doctor and, so I wasn’t sitting in there and trying to fill it out really quickly on their iPad [clinic-based questionnaire]. I felt like if I could go to the apps and fill out my symptoms right away, I wouldn’t forget. Having to wait for three weeks or whatever to fill out my symptoms makes it hard to remember. [46]

Furthermore, if patients experience symptoms at home but complete PROMs only when they visit the clinic, their symptoms might not be promptly identified, resulting in delayed management with potentially adverse outcomes [47]. Therefore, for all the abovementioned reasons, the home environment may provide the best place for symptom monitoring to improve health outcomes [48].

##### CMO 1.2: By Self-Reporting More Frequently, Patients Gain Better Awareness of Symptoms

Unlike reports completed in the clinic, self-reporting from home can be repeated more often, increasing patients’ awareness of their problems by focusing their attention on their symptoms and the pattern [49-53]. Several studies (3/61, 5%) exploring patients’ perceptions about ePROM systems found that patients agreed that it was easy to remember their symptoms when required to recall them at clinic encounters [50,54-56]. The following observations by Lee et al [57] and Richards et al [58] provide insights into how more frequent self-reporting might help patients:

I think those things will be helpful, and I’m not an organized person who writes something regularly about myself. But after doing this, I realized that I spent this week like this, and I was like this, and the change was like this. Yeah, it helped me a little, a little. [57]

And also...looking at the graph[s] and looking at my previous answers and seeing how they’ve changed, and interestingly as I said those euphoric first answers where everything was absolutely wonderful [when I was] still on tramadol and goodness knows what else. And then the reality of how I really am and then slowly seeing the recovery over that time has been very helpful. [58]

ePROM systems might also encourage self-monitoring through features such as graphic display of scores, time trends, and automated symptom alerts to patients [42,49,51,52,58-61], which may also provide reassurance to patients, particularly when presented with normative symptom information with prompts about when to reach out to their health care team [51]:

Basically it kept you where you were. What’s going on, knowing all your symptoms, keeping up with things and keeping up with you know the side effects. Yeah that was important to me. I’ve noticed now because I haven’t been using it. It’s hard to keep track of where I’m at what’s happening. [51]

I find it reassuring when I look at the graphs having completed it all...I find that it actually gives a pattern which reinforces how I feel about what has happened since the op. [58]

##### CMO 1.3: Remote Self-Reporting as a Communication Tool

Apart from increasing awareness of their health status, there is a suggestion that remote self-reporting might empower patients to express their concerns and communicate with their health care teams [50,55,61,62], which can be inferred from the observations of a clinician in a study by Cox et al [36] and patients in the studies by Gustavell et al [52] and Lee et al [57]:

It is something the patient has control of, and they are able to give you that information so you’re not waiting...they are in charge and able to give you information in between those phone calls. [36]

You just have to send in your report and then you get to talk to someone...I think you are more involved in care this way since you have your voice heard when you want. [52]

I think it’s very necessary that I report my symptoms through this app and then the app delivers the report to my doctor. And you know, informing my doctor about my symptoms may actually give me a feeling of stability. [57]

Several studies (6/61, 10%) support the notion that patients feel empowered to use real-time self-reporting to communicate concerns [45,48,50,54,63,64]. Falchook et al [45] found that patients reported 3 times more than weekly encounters when provided with ePROMs to report symptoms remotely, and 56% of reports were submitted on weekends and after hours. In the same study, all the patients who were surveyed agreed that reporting symptoms daily helped their clinicians manage them [45]. In a study by Wintner et al [48], 91% of home ePROM users considered it as a useful method to inform clinicians about their HRQOL compared with 65% of clinic ePROM users. Empirical studies that evaluated patients’ perceptions about how remote symptom self-reporting might support their care found that patients agreed that they felt more in control [7,50,54,63], and it improved discussion and communication with clinicians [7,54,63,64].

We found several possible mechanisms that may empower patients to communicate their concerns through remote self-reporting. First, the awareness that their reports are being sent to clinicians in real time may foster patients to view remote symptom reporting as a means to communicate directly with their clinicians. Patients may also consider it more important to communicate via symptom reporting when they have limited contact with their clinician between clinic encounters. One of the quotes from patient interviews in a study by Snyder et al [42] suggests that patients may view it as a direct way of communication with their clinician and not just for review in the clinic:

You need to go to your doctor with questions in writing, and it seems that this would be a vehicle to get those questions there. [42]

I am concerned where you say that you would not read it until the next appointment, you are not going to read it until I get there, what am I chopped liver or something? [42]

Patients’ request to add free-text and messaging functions to ePROMs and their appreciation of a system that is always available to report also suggests that patients intend to use real-time self-reporting as a platform to communicate with the clinician [37,41-43,48-50,52,61,65]. In the study by Denis et al [39], alerts were sent to clinicians whenever patients entered free-text comments in addition to the routinely scored symptoms; of the 43 email alerts triggered, 22 were owing to additional comments and the remaining were owing to symptoms breaching a predetermined threshold. A statement and a patient’s quote from the studies by Snyder et al [42,55] point to this mechanism:

Considering the option to add free text, patients like the direct communication, like their comments to inform the next visit, worried it will not be read. [42]

I think this is a good idea especially for people who tend to forget in between appointments what was going on and what they want to tell the doctor when they see him. This kind of takes care of the remembering for you. Being able to leave comments for the doctor from home is good too, especially since Dr. X told me that he saw them! That was nice. [55]

Second, by providing an electronic means to communicate their symptoms, ePROM systems may facilitate communication by reducing or eliminating patients’ hesitancy owing to uncertainty about whether symptoms warrant a call or worry about bothering their clinician [38,40,41,52,58,66]. Tailored feedback to patients, with alerts prompting when to contact clinicians, might also support communication by giving patients the legitimacy to contact clinicians [50,58,67,68] and, at other times, reassuring patients that they need not contact clinicians [41,60].

Third, patients may feel empowered not only because they view it as a means to communicate but also because by doing so, they feel monitored [48,49,52,53,65], reassured, and secure [37,39,41,51,58,65] owing to the perception that clinicians are watching over them through their reports, thus creating a feeling of being connected with their clinician between clinic visits and collaborating with their clinicians [39,52,65,66], which is illustrated in a patient’s quote from the study by Dawes et al [53]:

I felt when using the tablet as if I was in [the clinic]...it was IMPERATIVE to my health and recovery.

I was more aware of being monitored on a daily basis which in turn made me feel better connected to my health care providers.

##### CMOs 1.4: ePROMs May Become a Burden and a Reminder of Illness

We also found evidence for rival mechanisms that might negatively affect patients’ compliance with self-reporting. Although self-reporting may benefit patients by increasing their awareness of symptoms, at the same time, they must also confront their illness each time they self-report [49,50,66]. In a study by Egbring et al [69], 5 patients in the group using the mobile app withdrew to avoid being constantly confronted about their disease. Some patients declined participation in ePROM studies because they did not want to be reminded about their symptoms or their disease [43,66]. A quote by a patient reported in the study by Crafoord et al [49] provides more insight into when patients might perceive self-reporting as confronting and as a negative experience:

It has been very easy and convenient.... The app is easy to use...When you feel ill it is a security, but if you feel good it is a negative reminder that you are sick.

Crafoord et al [49] argued that patients might find it less meaningful to report when their symptoms are chronic than when new symptoms are emerging and tend to fluctuate. In situations where symptoms have become a part of patients’ lives or when interventions are unlikely to resolve them, ePROM reporting may be viewed more as a burden than adding any value to their care, as illustrated in a patient quote from the study by Snyder et al [55]:

It made me feel bad answering [sexual function] every time when the situation won’t ever change while I am on these medications so why keep asking about it? It just reminded me of that loss and made me feel bad every time.

Some clinicians even envisage that symptom reporting may lead some patients to become preoccupied with their illness as they are constantly reminded of it; this may particularly be relevant in the cancer survivorship scenario [66,67]. Therefore, whether patients perceive ePROM reporting as a negative or positive experience may be determined by the value that the ePROM reporting brings to patients’ overall care. In the context where ePROM reporting helps identify new symptoms and reduce symptom burden, it might provide patients with reassurance and security. However, when symptoms are not amenable to effective interventions, it might be viewed as a burden.

The relevance of the ePROM questionnaires to patients’ illness experience may also have a significant bearing on how patients perceive ePROM self-reporting [58]. If ePROMs are not relevant to patient’s health status and needs, they might perceive it as impersonal [48,55], negatively affecting their engagement with ePROM reporting [67,70], as illustrated by a patient’s quote:

Overall I don’t feel it was very helpful. It feels impersonal, just felt like extra paperwork that I had to do. I don’t know how useful the questions are. They only seemed to ask about things that we talk about during the appointments anyway. [55]

Even a callback from a clinician might only be appreciated if the patient perceives it as tailored to their needs, that is, if alerts and resulting callbacks were specific and meaningful to patients [37,59].

Basch et al [63] demonstrated a strong association between ePROM completion rate and patients’ perception about the usability, comprehension, meaningfulness, and utility of ePROMs. A Korean study [57] evaluated the differences in the characteristics of patients classified as adopters—patients who used the ePROM mobile app voluntarily at least once after installation in 7 days—and nonadopters of an ePROM mobile app. This study identified that adopters were more likely to report ease of use and ease of notifying symptoms to their clinicians than nonadopters. Adopters and those classified as having good compliance—patients who adopted the app and continued using it for 21 days with a reporting interval of approximately 7 days—were also more likely to report that the app helped them recognize their health and manage symptoms. In the qualitative part of the study, the themes identified for poor compliance were “reporting fatigue” and “app poorly reflecting their health status.” These 2 studies support the hypotheses that the relevance of ePROMs to patients’ situations and the perception that ePROMs enable the management of their symptoms are important for adherence with remote self-reporting.

##### Theory Testing for IPT 1

In the study by Shiroiwa et al [71], we found evidence to support our hypothesis that when patients report from home more frequently, it can capture patients’ health status in great detail, supporting CMO 1.1 and CMO 1.2. This Japanese study randomized patients between paper-based PROM completion in the clinic and ePROM completion at home. The ePROM group completed ePROMs more times, and both groups completed the EQ-5D-5L and the European Organization for Research and Treatment of Cancer Quality of Life Questionnaire–core questionnaire 30-item (EORTC QLQ-C 30). Although the adjusted mean difference in scores (EQ-5D-5L and EORTC QLQ-C30) at the same time points was equivalent between the paper-based and ePROM groups, there was a statistical difference between the 2 groups in quality-adjusted life days (QALDs) for EQ-5D-5L, with QALDs being low in the ePROM group. The low scores were explained by ePROM scores capturing the fluctuation in patients’ health status between chemotherapy when they reported from home and paper-based PROMs administered only in the clinic. Moreover, when the longitudinal score was plotted in the ePROM group, the trend followed the expected drop in scores a few days after chemotherapy and subsequent recovery before the next chemotherapy, which was not seen in the paper-based PROM group, suggesting that ePROMs were more accurate in depicting HRQOL after chemotherapy. However, the 2 groups did not show a similar difference in QALDs when it was calculated from EORTC QLQ-C 30. The authors argued that the lack of difference in QALDs based on EORTC QLQ-C 30 scores was because the questionnaire has a 1-week recall period, whereas EQ-5D-5L did not. Albeit speculative, if a 1-week recall period in a questionnaire can introduce recall issues, it supports our assumption that real-time reporting as symptoms occur could improve reporting accuracy by eliminating recall issues.

The evidence to indicate that remote self-reporting enhances patients’ symptom awareness and enables empowerment and better symptom monitoring was identified in a few studies. In the study by Bae et al [47], clinicians could better record symptoms when patients self-reported remotely, as demonstrated by a significant increase in the number and grade of the recorded adverse effects after patients were provided with smartphone-based self-reporting. The number of patients who perceived that they could thoroughly manage their symptoms or that reporting enabled them to notify their clinician about their symptoms increased but not significantly (CMO 1.3). However, it could also be argued that the increase in the adverse effects recorded was a result of improved clinicians’ awareness of the patient’s symptom experience—another plausible mechanism that is further explored in IPT 2. In another study, Kearney et al [72] evaluated whether mobile phone–based symptom self-reporting could improve symptom monitoring during chemotherapy. Patients were required to report twice daily between days 1 and 14 of each chemotherapy cycle, and the outcomes were assessed using a paper-based symptom questionnaire before cycles 2 to 5. There were less reports of fatigue but more reports of hand-foot syndrome in the group receiving the intervention. This discrepancy was attributed to the heightened symptom awareness induced by frequent reporting of the symptoms using the mobile phone, supporting CMO 1.2.

Similarly, when patients reported more frequently from home while being prompted by automated reminders, they generated more alerts to clinicians, enabling better symptom monitoring and treatment adherence in the studies by Graetz et al [44,46]. Patients in this study who completed the postintervention semistructured interviews attributed the better mental health scores to “feeling cared for” and the reduction in physical scores to more frequent use of the app prompted by reminders, which made them think about their symptoms more often, thus increasing the awareness of physical symptoms, which supports CMOs 1.1 and 1.2 [44]. The empowering influence of the ePROM intervention is also supported by observations from the study by Tolstrup et al [68]. Patients in the intervention group who received tailored feedback after submitting self-reports made more phone calls and unplanned visits in this study. Interestingly, the intervention group also had better HRQOL at 48 weeks; however, the ePROM intervention ceased at 24 weeks, indicating that increased awareness of symptoms and, perhaps, empowerment was enduring.

The importance of self-reporting frequency is also supported less directly by 3% (2/61) of studies that evaluated the effect of the cadence of ePROM self-reporting on symptom monitoring [73,74]. Daly et al [74] evaluated daily ePROM reporting in patients initiating anticancer treatment and observed that 45.1% of red alerts generated by completing ePROM reports were not preceded by yellow alerts in the previous 7 days. There was a 3-fold increase in acute events when red alerts were generated compared with no red alerts in the preceding 7 days. Therefore, less-frequent reporting might not only miss red alerts because they are not preceded by yellow alerts but also miss the opportunity to prevent adverse events. Another study by Innominato et al [73], with a small sample size, used exploratory analysis of longitudinal data and showed that 55.6% of severe symptoms might be missed when symptoms were sampled only weekly. Presuming that the immediacy of self-reporting when symptoms occur is important to accurately report symptoms without being affected by recall issues, it can be inferred from the abovementioned studies that frequent reporting may offer a better opportunity to identify symptoms and potentially prevent downstream adverse events.

##### Additional Contextual Influences for IPT 1

Apart from the general contextual factors described in CMO configuration that are thought to enable the mechanisms, individual patient characteristics might also be important to realize outcomes. Patients’ literacy and computer experience may moderate the benefits obtained through ePROM reporting. In a study evaluating the relationship between patient characteristics and the perceived value of remote self-reporting [63], high education and previous internet use positively correlated with usability but negatively correlated with meaningfulness or relevance and communication or actionability, which indicates that patients with low education and limited internet skills perceived more value from ePROMs. However, in other studies, the patient group with previous internet experience showed more adherence [54,75]. Nonetheless, in the RCT conducted by Basch et al [4] among patients undergoing chemotherapy and comparing ePROM intervention with usual care, computer-inexperienced patients obtained a greater benefit because the reduction in emergency department visits and hospitalizations were more pronounced in this group compared with computer-experienced patients. It should be noted that the computer-inexperienced patients were asked to report only at the clinic visits, whereas computer-experienced patients reported from home also in this study.

Patients’ level of self-reliance may also determine how much benefit they would derive from ePROM interventions [49]. On the one hand, patients may become dependent on ePROM system, showing less initiative to contact clinicians [76], as was seen in some studies in which patients did not contact the clinician even when automated feedback advised them to contact. This group of patients may prefer to avoid taking responsibility for deciding when to contact clinicians and would prefer their clinicians to initiate contact [76]. However, in contrast, self-reliant patients may want to exercise more control over their interactions with clinicians [49,52,60]. Patients may even feel compelled to alter their response to avoid triggering a clinician callback when they feel it is unnecessary [52,60].

Patient’s symptom burden may be a motivating factor to self-report and, perhaps, an indicator for obtaining more benefit from the intervention. Among patients with myeloproliferative neoplasms, Brochmann et al [43] found that the patients with disease subtypes with high symptom burden and low HRQOL completed ePROM reports more frequently, albeit not to a statistically significant degree. Similarly, Judson et al [77] found that patients undergoing chemotherapy with advanced-stage cancers showed a high likelihood of compliance, and the baseline Eastern Cooperative Oncology Group performance status did not affect compliance. However, Basch et al [54,75] found a variable effect of baseline performance status on adherence in patients receiving chemotherapy: performance status did not affect adherence in patients with lung cancer, but in patients with gynecological cancers, better performance status was associated with high ePROM system log-ins. Finally, Absolom et al [78] found that patients with better baseline physical well-being showed better adherence to self-reporting and benefited most from ePROM intervention. Therefore, although high symptom burden may portend high likelihood of adherence to self-reporting, patients’ poor performance status could offset the benefits of self-reporting.

We have summarized the program theory 1 about how remote symptom self-reporting could improve health outcomes and the factors that may moderate the outcomes in Table 2. Table 2 presents the program theories, statements about rigor, and literature sources.

**Table 2:** Program theories, statements about rigor and literature sources

Theory number	Program theory	Statement about rigor	Literature source for refining and testing
1	Remote self-reporting enables patients to recognize and report symptoms accurately through the following: Enabling reporting from a calm and self-reflective home environment Facilitating real-time reporting, thus reducing recall issues Fostering better symptom awareness and insights through frequent reporting and self-monitoring Empowering patients to communicate their problems by creating a feeling of being connected and providing legitimacy Patients may disengage from self-reporting, which may occur owing to the following reasons: ePROMs^a^ become a reminder of an illness ePROMs do not resonate with the patient’s situation The overall benefit from ePROM self-reporting may also be determined by the following additional contextual factors: Patients’ symptoms burden Patients’ computer or internet experience and self-reliance	Empirical studies adequately supported the CMO^b^ configurations; however, the overall rigor at the theory level was affected owing to the need for empirical studies to link specific mechanisms and outcomes directly.	Shiroiwa et al [71], Bae et al [47], Kearney et al [72], Graetz et al [44,46], Tolstrup et al [68], Innominato et al [73], Daly et al [74], Basch et al [4,54,63,75], Crafoord et al [49], Coolbrandt et al [76], Gustavell et al [52], Warrington et al [60], Brochmann et al [43], Judson et al [77], and Absolom et al [78]
2	Real-time feedback to clinicians promotes a proactive attitude to symptom management, enabling timely management of symptoms through the following: Providing a better picture of patients’ symptoms between clinic encounters Increasing clinician receptiveness and sensitivity to feedback through real-time feedback alerts EMR^c^ integration and usability (graphical display, highlighting key changes, and decision aids) encourage clinicians to use feedback reports through the following: Providing a context to interpret feedback reports Facilitating clinicians to view it similar to laboratory reports However, clinicians may trade off reviewing ePROM reports if the feedback reports and alerts are not perceived as specific and meaningful	Empirical studies adequately supported the CMO configurations, but the rigor at the theory level was affected by the lack of studies to test the link between mechanisms underpinning EMR integration and usability and health outcomes.	Simon et al [82], Cleeland et al [83], Mooney et al [81,85], Girgis et al [8], Egbring et al [69], Spoelstra et al [84], and Yount et al [86]
3	Timely feedback from clinicians through telephone interactions or e-interactions improves symptom management through the following: Reinforcing patients’ engagement with self-reporting by fostering a perception that clinicians are sensitive to their concerns Promoting early patient-centered interaction between clinic visits	The CMO configuration was supported by robust evidence with adequate rigor at the theory level.	Lee et al [57], Absolom et al [78], Cleeland et al [83], Mooney et al [81], Pappot et al [87], Greer et al [88], Mir et al [9], Zhang et al [89], Fjell et al [90], Maguire et al [79], Basch et al [4,7], Hough et al [91], and Denis et al [6]

^a^ePROM: electronic patient-reported outcome measure.

^b^CMO: context-mechanism-outcome.

^c^EMR: electronic medical record.

##### Rigor

Regarding the rigor of the evidence source, the CMO configurations were drawn from a large pool of empirical studies with varied methodologies, which were assessed to have adequate quality. Among the studies used to test whether the theories can be linked to health outcomes, none of the RCTs blinded the outcome assessors. In the studies by Kearney et al [72], Tolstrup et al [68], and Shiroiwa et al [71], the comparability of the participants in the 2 groups could not be ascertained because the baseline characteristics described did not include the literacy or computer experience of the participants. The outcome data could not be considered as complete owing to significant attrition or missing data in 3% of the studies [68,72]. The study by Bae et al [47], a prospective before-after study, did not account for participants’ general or digital literacy as a confounding factor in their analysis. Regarding rigor at the theory level, the CMO configurations were adequately supported by empirical studies; however, the process of determining whether health outcomes improved and whether they could be linked to the specific mechanisms required some assumptions and triangulation when the studies did not provide direct evidence to connect the mechanisms to health outcomes.

#### IPT 2

##### CMO 2.1: Promoting Proactive Symptom Management by Clinicians Through Real-Time Feedback of Symptom Reports

Real-time feedback of ePROM reports may promote proactive management of patients’ problems by clinicians, and there are several plausible mechanisms of action through which this may occur. First, real-time feedback may increase clinicians’ awareness of symptoms [50,62,67], permitting early identification of patients’ problems and prioritizing symptom management [50,55,62,67]. As remote monitoring allows more frequent reporting, the feedback of reports may provide deep insight and full picture of patients’ symptom experiences by providing far more information in the period between clinic encounters than symptom reports completed only in the clinic [66], as noted by a clinician in the study by Cox et al [36]:

You could end up with a really good diary of how things have been over a week which you could then use to manage their pain more appropriately.

Second, as the ePROM reports are available to clinicians in real time, they may allow clinicians to identify and manage problems early without needing patients to contact clinicians between clinic encounters [66,79]. Furthermore, through alerts that inform clinicians about significant events, real-time feedback may facilitate early intervention with great potential to improve health outcomes [76], as illustrated in the statement by Maguire et al [66]:

This anticipatory and preventative approach to care was also mentioned by HP03 [health care provider] as a good approach to having “infrastructure to manage it and monitor. And identify patients who are alerting and have early intervention to prevent deterioration in the symptom.”

The study by Maguire et al [80] explored the perceptions of nurses who participated in a study investigating ePROMs and found that 62% of the nurses considered that remote symptom reporting with clinician feedback enabled early detection of symptoms, and 50% felt that it resulted in the institution of timely management.

Third, feedback alerts may also increase clinicians’ responsiveness, which connotes clinicians’ participation in monitoring the feedback reports and responding to feedback. Augmenting clinicians’ responsiveness may be an important mechanism of action because clinicians’ responsiveness to feedback without alerts has been low [8,81]. Therefore, real-time feedback, with triggered alerts that prompt clinicians to respond, may change the clinician’s predominantly reactive approach to symptom management to a more anticipatory and proactive approach [51,65].

##### CMO 2.2: Electronic Medical Record Integration of ePROMs and Ease of Use Encourage Clinicians to Use the Feedback

Electronic medical record (EMR) integration of ePROM feedback reports has been regarded as an important element in ePROM programs. It may enable prompt review of ePROM reports, especially when ePROM data are captured and fed back in real time. Clinicians largely view ePROM interventions as beneficial for patient care but are also commonly concerned that these can increase their workload [50,75,80]. Real-time feedback and alerts can increase clinicians’ workload because the volume of information captured will be much larger than the clinic-only reporting, and they will be required to review the alerts [63,75]. In the Patient-Reported Outcomes to Enhance Cancer Treatment (PRO-TECT) trial, 47.3% of the nurses perceived that the alerts were too many [63]. Simon et al [82] reported that the number of nursing calls per patient within 30 days after surgery increased by 34% when ePROM self-reporting was available. Moreover, clinicians may be willing to trade off the benefits of using ePROMs if reviewing feedback reports consumes substantial time, particularly when the ePROM usability is not optimized. Biran et al [59] noted clinicians’ reluctance to use ePROM reports:

The clinicians reported not using symptom graphs and reports during the study due to the high volume of information that typically needs to be reviewed before and during a clinic visit and the lack of integration of the data into the EMR system and routine visit workflow.

Wu et al [61] also observed that lack of integration into EMR hindered the use of ePROM reports:

Having to log on to more than one site [eg, both EHR and PatientViewpoint] was viewed as a barrier. Several clinicians stated that they would be willing to sacrifice the graphical presentation of results to avoid the hassle of logging in to an additional system.

Therefore, EMR integration might encourage the clinician to use ePROM reports, overcoming their inertia in using it by reducing the time taken to view feedback reports through separate access and by serving as a reminder to check the reports akin to reviewing laboratory reports [42,50,55,61]. Furthermore, integration into EMR and workflow may foster an impression that ePROMs are integral to patients’ care as it may mirror clinicians’ clinical routine and enable clinicians to use it similar to laboratory reports [42]. Moreover, the impetus to use ePROM reports will be missing if the reports are not provided alongside other relevant clinical information because the ePROM reports might lack the necessary information to provide context to the ePROM scores, as suggested by Taylor et al [38] and by a clinician in the study by Snyder et al [55]:

I don’t think that the EPR information was helpful. It has no context. [55]

Health professionals found it difficult to interpret reports without prior knowledge of the patient, more detailed pain information and a full clinical history. [38]

Therefore, associating ePROM scores with patients’ clinical details and laboratory reports may provide clinicians with a better perspective about the ePROM scores in relation to the patient’s clinical situation, thus enabling interpretation and use of ePROM reports [42].

In addition, time trends with graphical displays of symptom scores [55,59] that emphasize important and meaningful changes in scores [42] that make feedback reports actionable and easily interpretable are also considered as enablers of the use of feedback reports [47,59,61]. The observation that alerts may lose their significance and not function as anticipated if they are not specific also emphasizes the importance of meaningful alerts [59]:

Staff members explained that it would be most useful to be alerted of newly emerging, severe, or worsening symptoms; however, the alert logic also frequently captured chronic and expected symptoms. In order to maximize clinical utility, they suggested the alert criteria be made more specific so that more actionable app alerts were generated.

Furthermore, poor specificity of the automated alerts may increase the volume of alerts and lead to the perception that symptom monitoring and alerts increase workload without a meaningful benefit [66,80].

##### Theory Testing for IPT 2

To test the theory that real-time feedback might promote clinicians’ awareness and improve health outcomes, we first examined the empirical studies that used alert-based feedback as the primary means of clinician feedback after symptom monitoring rather than a clinic review of ePROMs. Simon et al [82] analyzed the effect of introducing postoperative symptom tracking with automated risk-stratified alerts to the clinical team. Notably, the ePROM system did not present a summary report at the clinic visit; hence, any effect can be attributed to the alert-triggered proactive communication between the clinician and patient and the effect of symptom reporting itself on patients’ awareness of symptoms and in providing reassurance to patients about their symptoms (as per IPT 1). There was 22% reduction in the odds of urgent care center visits after introducing symptom tracking and 42% reduction in responders who completed at least 1 survey [82]. Overall, 3% (2/61) of the studies [81,83] used telephone-based daily or twice-a-week symptom reporting with threshold-based, real-time alerts to clinicians. However, only 2% of the studies showed improved outcomes [83], showing significant reduction in symptoms over time that breached a predetermined threshold. An important difference between these 2 studies was a relatively high proportion of clinician-initiated contact in the study [83] that showed improved outcomes compared with the study [81] that did not—60% versus 17.5%. Although the reason why alerts did not motivate clinicians to review and react to reports in the study by Mooney et al [81] is unclear, the difference in the clinician-initiated contact could explain the difference in the outcome, suggesting that systematic clinician feedback to patients can improve health outcomes, if clinicians respond to alerts. Evidence for the effectiveness of alerts was also seen in the study by Girgis et al [8]. This study showed that the number of emergency department visits, the primary outcome, was significantly low (by 33%) in the intervention group. Clinicians’ responses to alerts were informative in this study: only 32% of the passively provided feedback reports were reviewed, mostly by nurses (82%), but when the reports were associated with alerts, 44% of the reports were reviewed. The abovementioned studies support the notion that real-time feedback to clinicians can improve health outcomes, and alert-based feedback might be important to heighten clinicians’ responsiveness to feedback reports, which supports CMO 2.1.

In the studies that compared ePROM self-reporting with or without feedback to clinicians, patients who had their ePROM report feedback to clinicians fared better. Egbring et al [69] found that only the group completing daily reports via the mobile app with physician review of reports showed stabilization of functional activity over 3 visits. More distinct adverse events were reported in the app than in the questionnaire in both groups using the app, but this difference was highest in the group using the app with feedback to physicians. The authors postulated that mobile app–based reporting and physician feedback may have helped patients to precisely report symptoms, facilitated better communication, and improved physician awareness, which could have led to better symptom management. Similarly, Spoelstra et al [84] identified that symptom self-reporting via telephone-based interactive voice response (IVR) with symptom self-management toolkit for patients receiving oral chemotherapy was equally effective for decreasing symptom severity compared with symptom self-reporting with nurse feedback. However, the largest decrease in symptom severity scores was seen in IVR with nurse feedback for symptom management and adherence. The studies by Spoelstra et al [84] and Egbring et al [69] not only provide evidence to support that remote self-reporting enables better symptom reporting (IPT 1) but also suggest that when feedback reports are provided to clinicians, there is a demonstrable improvement in health outcomes, which can be taken to indicate that real-time feedback perhaps enabled better management of symptoms (CMO 2.1).

Regarding whether increasing the usability of ePROM feedback reports may influence health outcomes (CMO 2.2), there is direct evidence from the study by Mooney et al [85] and indirectly from Yount et al [86]. Mooney et al [85] conducted a second RCT after their abovementioned study failed to improve outcomes [81], aiming to overcome clinician inaction and prompt clinicians to intensify symptom management in the investigation arm by adding a guideline-based decision support system for the nurse receiving alerts. Although the study did not provide details about whether clinicians responded to more alerts compared with their previous study, unlike the previous study, it demonstrated significant reduction in symptom burden and the number of severe and moderate symptom days. The authors argued that providing guideline-based decision support prompted clinicians to intensify symptom management. The decision support system may have increased the usability of feedback reports, which supports the theory that ease of use is important for realizing outcomes through real-time clinician feedback. In the RCT by Yount et al [86], no difference in the symptom distress scores was seen between patients randomized to either symptom monitoring only (via IVR) or symptom monitoring with automated alerts. The authors suspected that the sheer number of alerts overwhelmed the clinicians, and the lack of guidelines to respond might explain the negative results. The intervention group also made more phone calls; nonetheless, contrary to what would be anticipated, they also had low satisfaction in their perception about having their concerns understood and whether they received adequate support and information. Therefore, poor specificity of the automated alert system may have a double negative effect: overwhelm clinicians with a large number of alerts and leave patients dissatisfied because they will not receive feedback about their symptom reports from clinicians. These 2 studies [85,86] suggest that improving the actionability of feedback reports might play a critical role in involving clinicians in using feedback reports, and the sensitivity and meaningfulness of the feedback reports are also vital (CMO 2.2).

The program theory 2 about how real-time clinical feedback might improve health outcomes and the factors that may affect it is summarized in Table 2.

##### Rigor

The data sources that contributed to the generation of the CMO configurations were largely of adequate quality. The quality appraisal of the empirical studies used for testing indicated the following potential for bias: in the study by Cleeland et al [83] and Yount et al [86], owing to incomplete outcome data; in the study by Spoelstra et al [84], the baseline symptom severity was significantly different between the groups, and it was not clear whether randomization was concealed; in the study by Egbring et al [69], it was not possible to ascertain the baseline comparability between the groups because the baseline characteristics described did not reveal the general or digital literacy level of the groups; and in the study by Mooney et al [81], the imbalance in the baseline characteristics was owing to significantly high number of women with breast cancer in the intervention group. Only 2% of the studies [69] blinded the outcome assessors. In the quasi-experimental study by Girgis et al [8], more patients belonged to a disadvantaged socioeconomic group or had stage 4 cancer in the matched controls compared with the intervention group, raising the possibility of risk of bias. The retrospective cohort study by Simon et al [82] fulfilled all the methodological quality criteria for nonrandomized studies.

We assessed the rigor at the theory level to be moderate for CMO configuration 2.1; however, for CMO configuration 2.2, there was a lack of evidence to link the mechanisms and improvement in health outcomes. As with program theory 1, the evidence sources that enabled testing CMO configuration 2.1 also did not provide a direct link between mechanism and outcome and required assumptions and triangulation between the sources.

#### IPT 3

##### CMO 3.1: ePROM-Enabled Telecommunication Reshapes Clinician-Patient Interactions

In this CMO configuration, we determined that ePROM-enabled, clinician-patient interaction could be a plausible mechanism underlying the effectiveness of ePROM interventions. There is a strong suggestion that clinician-patient interactions also ensure patients’ compliance with self-reporting, an important ingredient for the success of any ePROM program [47,57,61,70]. Observations in a few studies support this: when patients were aware that clinicians reviewed the reports, the perceived usability of the ePROM system increased [55]; patients who had alerts sent to clinicians were more comfortable with the system and were more likely to perceive it as easy to use compared with patients who reported without alerts to clinicians [83]; and patients valued callback from their clinician in response to self-reports and when their reports were included in the consultation at the clinic encounters [50,59,61]. Moreover, in the context of ePROM program with alert-based feedback to clinicians, most of the patient-clinician interactions may not occur in the clinic but between clinic encounters because these interactions are triggered by alerts generated by patients’ remote self-reports. Unlike clinic encounters, these triggered clinician-patient interactions are mostly conducted over the phone and directly in response to patients’ symptom reports. Therefore, they are bound to be more patient-oriented and foster patients’ perception that clinicians are sensitive and caring [79], as can be inferred from a patient’s observation in the study by Dawes et al [53]:

Knowing your doctor has access to you on a daily basis was extremely helpful. Invaluable, actually.

The doctors were really interested in learning what their patients are going through, which doesn’t always get reported in office visits.

Lack of timely clinician feedback could discourage patients from self-report [47,61,75]. In essence, this means that ePROM programs that are integrated into patient care so that the PROM reports are systematically reviewed and responded to by clinicians are likely to demonstrate better patient compliance and, therefore, better outcomes [70,75]. Furthermore, patients’ adherence to self-reporting and clinicians’ responsiveness to feedback may reinforce each other through clinician-patient interactions: as systematic feedback from clinicians improves patients’ adherence to self-reporting, it captures patients’ illness experience in great detail, providing clinicians more opportunities to interact, further improving patients’ experience with self-reporting and adherence. Therefore, ePROM program could enable a fundamental shift in how patients and clinicians interact, beyond the bounds of the clinic, to predominantly patient-centered telephone interaction or e-communication between clinic encounters.

##### Theory Testing for IPT 3

In 3% of the studies, we found evidence to support our assumption that clinician feedback reinforces patients’ adherence [57]. The first study evaluated the factors that led to adopting and complying with an ePROM app—all participants in their qualitative interviews agreed that clinician feedback would reinforce their compliance [57]. The second study by Absolom et al [78] provided more definitive evidence for clinician responsiveness reinforcing patients’ compliance with self-reporting. In this study, among the patients who were assigned to weekly symptom monitoring and real-time feedback with alerts to the clinician using Electronic Patient Self-Reporting of Adverse-Events: Patient Information and Advice (eRAPID), there was a statistically significant association between patients’ adherence to reporting and clinicians’ use of eRAPID: for every 1% increase in times the clinicians used eRAPID, the odds of patients being in the high adherence group increased by 1%.

Whether clinician-patient interaction initiated by clinicians in response to ePROM completion can influence health outcomes can be understood by comparing the previously mentioned studies by Cleeland et al [83] and Mooney et al [81]. Although both studies used telephone-based, daily ePROM reporting with threshold-based, real-time alerts to the clinician, only Cleeland et al [83] showed significant reduction in symptoms. The difference between these 2 studies was the number of clinician-initiated contacts: 17.5% versus 60% in favor of the study by Cleeland et al [83].

Presuming that one of the important underpinning mechanisms by which ePROM interventions might improve health outcomes is by promoting direct clinician-patient interaction; we postulated that ePROM interventions that lack the architecture to promote relevant clinician-patient interactions might not achieve the desired outcomes. We investigated this by juxtaposing studies that enabled clinician-patient interaction and those that did not. The study by Pappot et al [87] and Greer et al [88] are 2 studies that neither include automated alerts in the design of the ePROM intervention nor mandate clinician review of real-time ePROM reports. Both failed to show any improvement in the evaluated outcomes. In both these studies, clinician-patient interaction in response to symptom reporting was lacking in contrast to other studies that showed a positive impact of remote symptom monitoring in a similar patient population. In comparison, studies that showed improvement in the health process–related or health-related outcomes enabled clinician-patient interactions in many different ways, for example, by using nurse navigators to receive all symptom alerts, advising patients using decision support algorithms [9], or mandating that ePROM app–triggered email alerts were followed up by phone calls immediately or on the same day [89,90].

It is also informative to interrogate clinician-patient interactions in studies that showed improved health process–related or health outcomes. In the European, multicenter, randomized trial using Advanced Symptom Management System [79], 95% of amber alerts and 85% of red alerts were reviewed within the mandated time frame; in the study by Basch et al [4], telephone counseling was initiated by nurses in response to 77% of email alerts; in the study by Hough et al [91], clinical pharmacists contacted patients 81% of the times when they were deemed to require real-time review triggered by the breach of predetermined threshold; and in the PRO-TECT trial [7], immediate nurse interventions, which included telephone discussion, self-management advice, and medication prescriptions, ensued in response to 59.1% of alerts. Furthermore, in the study by Denis et al [6], alert-based feedback to clinicians prompted unscheduled visits in 58.3% of patients compared with 24.6% in the usual care arm, which presumably led to the detection of 72.4% of the lung cancer relapses between clinic visits compared with 32.5% in the usual care arm. These trials that showed a positive impact on health outcomes demonstrate that the ePROM interventions enabled direct telehealth interaction between the patient and their health care team much more efficiently compared with the studies that failed to improve health outcomes, which substantiates, although indirectly, that clinician-initiated interaction may be vital for realizing improvement in health outcomes by facilitating early identification and management of symptoms.

The program theory 3 about how clinician-patient interaction might improve health outcomes is summarized in Table 2.

##### Rigor

Several data sources of adequate quality supported CMO configuration 3.1. The risk of bias in the abovementioned RCTs, contributing to the testing of program theory 3, concerns the lack of blinding in all the studies (n=21) and significant attrition and missing outcomes in the studies by Basch et al [4], Greer et al [88], and Absolom et al [78]. Although missing values were imputed and sensitivity analysis was performed in the study by Absolom et al [78], most missing reports were from patients with worse physical well-being. The comparability of baseline characteristics between the arms could not be ascertained in the studies by Mir et al [9] and Pappot et al [87] because the former study did not report the general or digital literacy of the participants and the latter reported none of the baseline characteristics. The rigor of program theory 3 may be lessened at the theory level as it was supported by predominantly indirect evidence; however, the triangulation from different sources has provided sufficient rigor.

## Discussion

### Overview

The program theories (Table 2) developed in this realist synthesis describe the key mechanisms underpinning an ePROM intervention and the contextual factors important for improving health outcomes in routine cancer care. This study contributes to bridging the current knowledge gap regarding which elements in ePROM interventions are important and how they enable improved health outcomes. The 3 postulated program theories make explicit the mechanisms that underpin how each of the core elements of an ePROM intervention—remote real-time self-reporting, real-time feedback to clinicians, and the ensuing clinician-patient interactions—produce the desired outcomes. The following part of the discussion briefly describes and contextualizes the main findings with respect to each core element of ePROM intervention and their implications for practice.

### Real-Time Remote Symptom Monitoring

#### Main Findings

Real-time remote symptom monitoring through ePROMs could improve health outcomes by increasing symptom awareness and empowering patients to communicate with clinicians and report their symptoms accurately (program theory 1). Being able to self-report in real time from a home environment is a key element in ePROM intervention and is consistent with the emerging concept of ecological momentary assessment (EMA) that is increasingly advocated to improve the assessment of a patient’s symptom experience [92,93]. EMA is based on the premise that repeated measurement of patients’ experience in their natural environment captures the illness experience in real time, arguably providing a better assessment of patients’ illness experience [94]. By enabling real-time symptom monitoring and high cadence of measurement, ePROM interventions could use the principle of EMA to capture patients’ symptom experiences better. However, this will demand a stringent level of adherence to self-reporting from patients. Therefore, whether real-time symptom monitoring will produce the desired outcomes will ultimately be determined by patient engagement with real-time remote self-reporting.

#### Implications for Practice

To promote and maintain engagement with real-time self-reporting, ePROMs should be tailored to patients, so that they see value in it. On the basis of the contextually important factor identified in this review, ePROMs should be tailored to individual patient characteristics and illness trajectory. Regarding patient characteristics, patients who lack computer experience generally show lower willingness and adherence to ePROM self-reporting than patients with computer experience [54,75,77,95]. However, patients with low digital literacy might benefit from ePROM self-reporting, perhaps through the empowerment provided by the ePROM interventions [4]. Therefore, ePROM programs should endeavor to identify patients who are disadvantaged owing to lack of computer experience or digital access and bring them on board through targeted education and by enabling access to essential ePROM hardware and software, which will ensure that ePROM self-report is within reach for patients who are likely to benefit the most.

Patients’ illness trajectory is another important consideration in tailoring ePROM interventions. High symptom burden portends better adherence to ePROM self-reporting and is, perhaps, a marker for deriving more benefit from ePROM interventions. In this instance, better adherence could relate to patients’ perceived value in ePROM self-reporting—patients with higher symptom burden and fluctuating symptoms may see more value in adhering to self-reporting than patients with chronic and stable symptoms. Early identification and management of symptoms through ePROM symptom monitoring may also matter more for patients at high risk for developing problems owing to high cancer burden or active cancer treatment or when they are at high risk for cancer progression. However, this may not be true for patients with poor performance status owing to advanced cancer because their goals of care could be different, and frequent ePROM self-reporting may become a burden. Therefore, the intensity of ePROM self-reporting, tailored feedback, and ePROMs should be made to reflect and adapt to patients’ cancer illness journey, with more intensive symptom monitoring provided to patients at high risk for adverse events while on active treatment and modified as patients’ performance status declines and goals of care change. For patients undergoing follow-up or surveillance, more targeted monitoring, with emphasis on ePROMs that are most relevant to their illness, will be more meaningful. In this regard, patients should also be provided with opportunities to have a say in their symptom monitoring through shared decision-making based on their goals of care. However, ePROM self-reporting in real time will not be meaningful to patients if ePROM reports are not promptly reviewed by their clinicians.

### Real-Time Clinician Feedback

#### Main Findings

Real-time clinician feedback is hypothesized to give clinicians a better picture of patients’ illness experiences and heighten their responsiveness to ePROM feedback reports (program theory 2). Clinicians’ engagement will be crucial, as feedback must be reviewed and responded to promptly, but it will be practically impossible to review every feedback in real time. Alert-based feedback could circumvent this problem to an extent and could bolster clinicians’ responsiveness to feedback by focusing attention on the most important ePROM feedback reports; however, other factors that enable the usability of feedback reports, such as integration into EMR and clinicians’ workflow, and ensuring that feedback is meaningful and actionable are also equally important. Clinician engagement with reviewing and responding to feedback directly influences symptom monitoring, as timely feedback will not only determine patient compliance but could also reinforce their adherence to real-time self-reporting (program theory 3).

#### Implications for Practice

The implication of program theory 2 is that clinician feedback should be readily actionable and meaningful; otherwise, clinicians are less likely to use the feedback reports. Alert-based feedback can filter the ePROM reports for the most significant changes in ePROM scores, making the feedback more meaningful and effective in identifying patient issues. However, an emerging challenge in ePROM interventions is processing the large volume of ePROM reports into actionable data and generating timely and meaningful alerts. ePROM programs use different methods, including software modeling and algorithms, to process the data and improve the sensitivity and specificity of the alerts [93]. Regardless of the methods used, ePROM programs should be able to adjust the software modeling and algorithms to optimize the system to capture the most meaningful alerts consistent with patients’ goals of care and adapt as they change. In the future, the use of artificial intelligence in processing ePROM data is anticipated, which will create opportunities to further enhance the ability to fine-tune the ePROM systems in real time.

Nonetheless, real-time symptom monitoring is bound to disrupt existing practice and challenge health care systems because all the alerts and significant feedback reports must be responded to promptly. It will be demanding on the resources particularly when health services implement ePROMs at full scale. Moreover, oncologists or the patients’ primary treating physicians may not always have the time or the willingness to review feedback in real time, which may require health systems to explore alternate models of care [38].

Different models of care have been used in clinical trials. In some studies, oncologists reviewed the feedback reports, and in others, a nurse-led model was used to review and respond to feedback reports [7,9,78]. Girgis et al [8] noted that when ePROM reports were available to nurses and oncologists, nurses reviewed most of the reports, significantly more than the oncologists. In a study that evaluated the perceived clinical relevance of real-time symptom monitoring among clinicians, nurses rated the relevance higher than oncologists [76]. Hence, apart from easing the time pressures on oncologists, a nurse-led model may also tap into the differences in how nurses and oncologists engage in responding to ePROM feedback. Moreover, in all the studies in this review (n=61), the ePROM interventions were hospital-centered and the primary care physicians (PCPs) played no part. Sharing ePROM data with PCPs will be important for the comprehensive care of patients and the sustainability of ePROM interventions if they were to be widely implemented in a health care system. PCPs may even take the lead in receiving and responding to ePROMs, particularly in cancer survivorship and palliative care settings and in health systems where PCPs play a large role in the care of patients in the community. However, how ePROM interventions function in a health care model involving or built around PCPs is largely unaddressed. Ultimately, the health service implementing ePROM interventions will need to evaluate and decide on the most suitable model of care determined by the attributes of the health service.

### Clinician-Patient Interaction

#### Main Findings

In program theory 3, we explored the mechanisms that may be activated through the clinician-patient interactions initiated upon ePROM report completion. By enabling real-time, feedback-triggered communication, which might largely involve telecommunication, ePROM programs promote patient-clinician interactions outside the clinic environment. Unlike a routine clinic encounter, these interactions could facilitate more patient-centered interactions as these are directly linked to issues that patients raise through ePROM self-reporting. Therefore, by promoting clinician-patient interactions between clinic appointments, ePROM interventions could potentially alter the traditional model of clinic-based interactions and create opportunities for more proactive and timely patient-centered interactions.

For ePROM programs to enable clinician-patient interactions, they should equally promote patients’ active participation in self-reporting and foster clinicians’ engagement. A systematic review of feedback reports by clinicians and the ensuing clinician-patient interaction will not only reinforce patients’ engagement (program theory 3) but can also reinforce clinicians’ engagement when clinicians perceive value in such interactions for improving patient care [96]. Therefore, the quality of patient-clinician interactions could have a knock-on effect on promoting patient and clinician involvement alike and underscore the success of an ePROM intervention in realizing health outcomes.

#### Implications for Practice

Although providing timely feedback to patients is important for them to see value in ePROM self-reporting and sustain their adherence to self-reporting, it will be impractical for clinicians to provide real-time feedback around the clock. Hence, the first implication of program theory 3 is that ePROM interventions should explore ways to provide meaningful feedback to patients without placing undue burden on clinicians. A method that ePROM interventions have used is automated tailored feedback to patients for low-grade symptoms, often coupled with education and resources for self-management, allowing clinicians to focus on more severe symptoms. The study by Pusic et al [97], which serves as an example, successfully replaced intermediate-level symptom alerts to clinicians with automated patient feedback. In this RCT involving patients who had ambulatory surgery, in the control arm, both yellow and red alerts were sent to clinicians, whereas in the intervention arm, yellow alerts were replaced with automated, normative feedback to patients. This study found no difference in the urgent care visits between the 2 arms, suggesting that this method can be used to reduce workload without affecting health outcomes.

The second implication for the ePROM programs is that it will be important to consider how clinician-patient interactions—interactions could be either clinician initiated or patient initiated—are tailored to patients with differing needs. Clinician-initiated interactions are triggered by feedback alerts sent to clinicians, whereas patient-initiated interactions rely on motivating patients through tailored feedback to contact the health care team. Some studies enabled both directions of initiating interactions [4,78]. However, patients with low computer literacy and self-motivation might benefit more from the former. In contrast, the latter may be more suited for patients with high education, computer experience, and self-motivation.

The abovementioned point can illustrated by comparing the studies by van der Hout et al [98] and Basch et al [63]. In the RCT by van der Hout et al [98,99], survivors of cancer in the intervention group used Oncokompas, an app for HRQOL self-monitoring with tailored feedback and self-management education; patients received tailored feedback about when to contact clinicians, but the responsibility to contact rested on the patients. In contrast, in the study by Basch et al [63], the alerts were sent in real time to clinicians who initiated the interactions and hence did not require patients’ initiative to make contact. van der Hout et al [98] noted a large effect of the intervention on HRQOL in patients with low to moderate self-efficacy and those with high personal control and high health literacy, contrasting with the studies by Basch et al [63] that showed high perceived benefit for patients with low education and computer experience [63] and better health-related outcomes in patients with low computer experience [4].

Moreover, encouraging patients to interact with their health care team by providing automated feedback and advice about when to make contact may not work uniformly. In the study by Tolstrup et al [68,100], it was noted that, when prompted, just over a dozen patients made approximately half the phone calls, which suggests that not all patients will be willing to contact their health care team [100]. Nonetheless, it is plausible that patients with high self-motivation and literacy may benefit equally just from automated tailored feedback—that is, a less resource-intensive intervention. Therefore, ePROM programs need to be able to tailor how feedback is handled through a process of shared decision-making with the patients, taking into consideration the available resources.

### Future Studies

The following areas of research could inform the design and implementation of ePROM intervention in the future. It is worth exploring how to assess and identify patient subgroups with differing levels of self-motivation and self-efficacy, which could inform how ePROM interventions can be tailored to patients who may require more direct clinician feedback and those who might do well with just automated feedback with resources for self-management and less clinician involvement. Another area of research that could inform the incorporation of ePROM intervention into routine cancer care is the evaluation of different models of care pertaining to how ePROM feedback reports are integrated into the workflow within a health service and sharing of ePROM data with the wide health care team including PCPs and allied health professionals. It will also be important to evaluate how patients respond to different models of care and whether that matters for their perception about ePROM reporting as integral to their care and adherence. As ePROMs are increasingly used to follow-up patients after their treatment and in cancer survivorship care—complimenting and even partially replacing face-to-face visits in the clinic—it will be important to address the contextual factors and mechanisms that might underpin patients’ long-term adherence to telemonitoring and develop program theories specifically about how ePROM telemonitoring can be sustained over a long term in practice.

### Strengths of This Study and Comparison With Previous Studies

To the best of our knowledge, this is the first realist synthesis about how ePROM interventions can improve health outcomes in the cancer care setting. The strength of this review lies in its focus on the salient components of modern ePROM interventions, mechanisms that underpin the effectiveness of these components in improving health outcomes, and factors that may moderate the realization of the outcomes. It is, therefore, easy to conceptualize the practical implications of incorporating different components in the architecture of the ePROM intervention and how the components may interact. This realist synthesis differs in important aspects from those performed hitherto. The realist syntheses by Flynn et al [22] and Greenhalgh et al [20,21] were broad in scope, addressed PROMs in health care in general, and included studies evaluating ePROM and non-ePROM intervention in their syntheses. Flynn et al [22] reported IPTs that explored how PROMs are used in health care settings. The theories in the realist syntheses by Greenhalgh et al [20,21] were intended to explain how ePROMs improved communication and patient care. Although the theories put forth by Flynn et al [22] and Greenhalgh et al [20,21] are overarching theories that apply to any PROM intervention and have some commonalities with our theories, they were not intended to capture the mechanisms that underpin how ePROM interventions might improve health outcomes when used as a remote symptom monitoring tool. Therefore, the theories we have postulated can be considered more specific to the context of cancer clinical care and provide insights about the plausible mechanisms for the effectiveness of ePROM interventions in improving health outcomes, particularly when used as an eHealth intervention.

### Limitations

We recognized the following limitations in this realist review. As we focused on the mechanisms that operate through remote self-reporting and real-time feedback, we did not scrutinize other elements often used in ePROM programs, such as tailored patient feedback. Therefore, there might be mechanisms related to other elements in ePROM architecture that were not explored in depth in this review. We did not investigate all the variations in the architecture of the ePROM program and how they may shape the outcomes. For example, ePROM programs are increasingly used as a vehicle for digital interventions such as self-management education. We did not explore how these additions affect the operation of ePROM intervention as it was beyond the scope of this review. Finally, we did not explore how barriers to eHealth interventions, such as data security and privacy issues, moderate the realization of the outcomes.

### Conclusions

This realist synthesis articulates the key mechanisms behind how ePROM interventions can improve health outcomes in routine cancer care: empowering patients to report symptoms accurately and communicate with clinicians through remote self-reporting, promoting proactive management of symptoms by providing a better picture of patients’ illness experience through real-time feedback to clinicians, and facilitating clinician-patient interactions between clinic encounters. Our synthesis hypothesizes that ePROM interventions are more likely to improve health outcomes by engaging patients with remote self-reporting; encouraging clinician involvement through alert-based, real-time feedback; EMR integration; and improving the usability of feedback reports. Our findings suggest that the implicit mechanisms reinforce each other and may fundamentally reshape clinician-patient interaction between clinic encounters. Future studies should explore how patient characteristics moderate the benefit of ePROM interventions, which could inform how ePROM interventions can be personalized to patients’ needs.

## Appendix

Multimedia Appendix 1 Literature search and inclusion and exclusion criteria.

Multimedia Appendix 2 Data extraction and appraisal sheet.

Multimedia Appendix 3 Characteristics of the included studies.

Multimedia Appendix 4 Quality appraisal using Mixed Methods Appraisal Tool.

## Abbreviations

CMO: context-mechanism-outcome
EMA: ecological momentary assessment
EMR: electronic medical record
EORTC QLQ-C 30: European Organization for Research and Treatment of Cancer Quality of Life Questionnaire–core questionnaire 30-item
ePROM: electronic patient-reported outcome measure
eRAPID: Electronic Patient Self-Reporting of Adverse-Events: Patient Information and Advice
HRQOL: health-related quality of life
IPT: initial program theory
IVR: interactive voice response
PCP: primary care physician
PROM: patient-reported outcome measure
PRO-TECT: Patient Reported Outcomes to Enhance Cancer Treatment
QALD: quality-adjusted life day
RCT: randomized controlled trial
