# Supplementary material for: Assessing Patient-Reported Outcomes in Routine Cancer Clinical Care Using Electronic Administration and Telehealth Technologies: Realist Synthesis of Potential Mechanisms for Improving Health Outcomes
Source: J Med Internet Res. 2023 Nov 28;25:e48483. doi: 10.2196/48483 (PMC10716761; doi:10.2196/48483)
Supplement: Multimedia Appendix 1 [file jmir_v25i1e48483_app1.docx]

**Literature search, inclusion and exclusion criteria**

Ovid MEDLINE search: formal search

|  | **Results** |
| --- | --- |
| 1 | Patient Reported Outcome Measures/ or ("patient-reported outcome measure*" or "patient-reported outcome measure*").mp. |
| 2 | (("patient* report*" or patient-reported* or patient-specific* or "patient specific" or patient-relate* or self-report* or "self-report*" or self-assess* or self-assess*) and (outcome* or measure* or assess* or evalut* or symptom*)).mp. |
| 3 | (PRO or PROs or PROM or PROMs or PRM or PRMs).mp. |
| 4 | (ePRO or ePROs or ePROM or ePROMs or ePRM or "electronic patient-reported outcome*" or "electronic patient-reported outcome*" or "electronic patient-reported measure*" or "electronic patient-reported measure*").mp. |
| 5 | (exp Patient Outcome Assessment/ or Outcome Assessment, Health Care/ or "patient outcome".mp. or "outcome assessment".mp.) and (patient satisfaction/ or patient preference/ or "patient* satisfaction*".mp. or "patient* preference*".mp.) |
| 6 | (exp Surveys/ and Questionnaires/) or survey*.mp. or questionnaire*.mp. or interview*.mp. or "self-report*".mp. or self-report*.mp. or measure*.mp. or instrument*.mp. or scale*.mp. or tool*.mp. or construct*.mp. |
| 7 | 5 and 6 |
| 8 | electronic health records/ or health information exchange/ |
| 9 | (ehealth or e-health).mp. |
| 10 | (mhealth or m-health or "mobile health*" or web-based* or ((web* or mobile*) adj5 based*) or mobile-based*).mp. |
| 11 | exp Telemedicine/ |
| 12 | ((electron* adj5 (record* or monitor*)) or telemonitor*).mp. |
| 13 | (digital* adj5 (follow-up or care*)).mp. |
| 14 | exp Neoplasms/ |
| 15 | (tumour* or tumor* or neoplas* or malignan* or oncolog* or metastas* or cancer*).mp. |
| 16 | internet.mp. or Internet/ or *Internet-Based Intervention/ |
| 17 | Mobile Applications/ or mobile.mp. |
| 18 | 1 or 2 or 3 or 7 |
| 19 | 8 or 9 or 10 or 11 or 12 or 16 or 17 |
| 20 | 14 or 15 |
| 21 | 4 or 13 |
| 22 | 18 and 19 and 20 |
| 23 | 20 and 21 |
| 24 | 22 or 23 |

Ovid MEDLINE search: Focused search

| 1 | Patient Reported Outcome Measures/ or ("patient-reported outcome measure*" or "patient-reported outcome measure*").mp. |
| --- | --- |
| 2 | (symptom* or "health status" or "adverse event*" or well-being or functional).mp. |
| 3 | (PRO or PROs or PROM or PROMs or PRM or PRMs).mp. |
| 4 | (ehealth or e-health).mp. |
| 5 | (mhealth or m-health or "mobile health*" or web-based* or ((web* or mobile*) adj5 based*) or mobile-based*).mp. |
| 6 | exp Telemedicine/ |
| 7 | ((electron* adj5 (record* or monitor*)) or telemonitor* or "remote monitor*" or "real-time monitor*").mp. |
| 8 | (digital* adj5 (follow-up or care*)).mp. |
| 9 | exp Neoplasms/ |
| 10 | (tumour* or tumor* or neoplas* or malignan* or oncolog* or metastas* or cancer*).mp. |
| 11 | internet.mp. or Internet/ or *Internet-Based Intervention/ |
| 12 | Mobile Applications/ or mobile.mp. |
| 13 | ("clinical outcome*" or "clinical impact" or "clinical implication*" or "clinical utilit*" or "clinical relevance" or "clinical benefit*").mp. |
| 14 | (survival or progression or "treatment outcome*" or compliance or adherence or "adverse effect*").mp. |
| 15 | 1 or 2 or 3 |
| 16 | 4 or 5 or 6 or 7 or 8 |
| 17 | 9 or 10 |
| 18 | 13 or 14 |
| 19 | 15 and 16 and 17 |
| 20 | 19 and 18 |

Inclusion and exclusion criteria

| Inclusion | Exclusion |
| --- | --- |
| Formal literature search | |
| - Studies evaluating ePROM intervention with remote ePROM reporting and electronic feedback to clinicians. - Studies about clinician and patient perceptions of how ePROM interventions supported clinical care. - Studies in adult cancer patients (>18yrs of age) and clinical care settings. - Any study design: qualitative and quantitative and mixed-method studies. | - Studies using only paper-based PROM. - Studies using ePROM intervention without remote ePROM reporting and electronic feedback to the clinician. - Studies in children. - Studies using ePROM as an outcome measure to evaluate an intervention. - Studies evaluating only psychometric properties of ePROM. |
| Focused search | |
| - Studies evaluating ePROM intervention as a symptom-monitoring tool in cancer clinical care. - Include health outcomes or health-related process outcomes: adverse events, symptom severity, QOL, survival or progression-free survival, unplanned health care utilisation (hospital or emergency department admission) and treatment compliance. - Studies in adult cancer patients. - Comparative effectiveness studies: experimental and quasi-experimental studies, analytical observational studies. | - Studies in children. - Studies using ePROM as an outcome measure to evaluate an intervention. - Studies not reporting health outcomes or health-related process outcomes. |
