# Supplementary material for: Assessing Patient-Reported Outcomes in Routine Cancer Clinical Care Using Electronic Administration and Telehealth Technologies: Realist Synthesis of Potential Mechanisms for Improving Health Outcomes
Source: J Med Internet Res. 2023 Nov 28;25:e48483. doi: 10.2196/48483 (PMC10716761; doi:10.2196/48483)
Supplement: Multimedia Appendix 2 [file jmir_v25i1e48483_app2.docx]

| Data Extraction and Appraisal Sheet  Title:  First Author/year:  Type of article (quantitative/qualitative/mixed/review/others):  Reviewer:  Summary of the paper:  Describe the intervention and components (ePROM qualities/features/design):  Methodology, outcomes and results: |
| --- |
| What is the contribution to the theory areas?   1. Provider feedback and engagement 2. Patient feedback and engagement 3. Clinician-patient interaction |
| Describe the connection(s) between the outcomes and the process (C+M=0): |
| Describe any unintended (positive or negative) impacts and their mechanism link to the outcomes: |
| Appraisal |
| Relevance (Hight / Low):  Rigour  Mixed Methods Appraisal Tool:  1. Qualitative  2. Quantitative randomized controlled trials  3. Quantitative non-randomized  4. Quantitative descriptive  5. Mixed methods  Further appraisal required? |
