# Supplementary material for: Assessing Patient-Reported Outcomes in Routine Cancer Clinical Care Using Electronic Administration and Telehealth Technologies: Realist Synthesis of Potential Mechanisms for Improving Health Outcomes
Source: J Med Internet Res. 2023 Nov 28;25:e48483. doi: 10.2196/48483 (PMC10716761; doi:10.2196/48483)
Supplement: Multimedia Appendix 3 [file jmir_v25i1e48483_app3.docx]

**Characteristics of included studies**

|  | Author (Year) | Population | Intervention | Frequency of PROM | Components of the intervention | Outcomes | Key findings |
| --- | --- | --- | --- | --- | --- | --- | --- |
|  | Qualitative studies | | | | | | |
| 1 | Snyder et al [42] (2009) | 20 patients with breast or prostate cancers and 7 HCPs | PatientVie-wpoint (web-based) | - | ePROM self-reporting, feedback to HCPs with a graphical view, and integrated into EMR | Developing prototype website and  usability testing | Patients were more interested in change over time, free text options for direct communication, and favored questions about issues not normally discussed in the clinic.  HCPs appreciated the guidance on interpretation, emphasizing key findings and meaningful change over time, and integration into clinical routine. |
| 2 | Duman-Lubberding et al [67] (2015) | HCPs | OncoKompas (web-based) | - | ePROM self-reporting, tailored feedback to patients with advice on self-management and when to contract HCPs, and feedback to HCPs during clinic encounters (optional) | HCP perspectives | OncoKompas provided better awareness and a complete picture of patients’ symptoms and their severity; a tool to prioritize patients’ problems during a consultation. HCPs anticipated patient empowerment and increased engagement. HCPs were concerned that patients may obsess about their illnesses. It may remove patients’ responsibility in contacting HCP but raises concern that it may reduce motivation to seek help. |
| 3 | Wu et al [61] (2016) | 42 patients with breast or prostate cancer  and their HCPs (n=13) (sub-study in an RCT) | PatientVie-wpoint (web-based) | Before clinic visits | ePROM self-reporting (EORTC QLQ-C30, PROMIS, and supportive care needs survey), integrated into EMR, and available to HCPs at the clinic encounters | Quality improvement study | Patient perspectives: able to identify issues at home which might be otherwise missed; value diminished if HCP did not use the reports; valued symptom tracking feature.  HCP perspectives: preferred graphs; wanted a clear description of the meaning of the scores; viewed EMR non-integration as a barrier. |
| 4 | McCann et al [37] (2009) | 53 patients with breast, lung or colorectal cancer starting chemotherapy | ASyMS (mobile phone-based) | Days 1–14, morning, evening and ad hoc | ePROM self-reporting, real-time feedback to HCPs, risk-stratified alerts (pager) to HCPs, and tailored feedback to patients with self-management advice | Patient perspectives | 36 patients completed the post-study questionnaire.  The real-time HCP feedback and alert facility made patients feel secure and monitored. Patients felt reassured by using the system. |
| 5 | Richards et al [58] (2021) | 29 patients who had upper gastrointestinal cancer-related surgery | eRAPID (Web-based) | Twice weekly (1^st^ week) and weekly (8 weeks) post-discharge | ePROM self-reporting, tailored feedback to patients with self-management advice, real-time feedback to HCPs with graphs, automated email alert, and integrated into EMR | Patient perspectives | Key themes: reassurance and empowerment.  Reassurance through symptom tracking progress, understanding recovery, reducing the feeling of isolation, easing anxiety about symptoms, and prompts to contact HCP.  Empowerment through tailored advice on the management of symptoms. |
| 6 | Warrington et al [60] (2019) | 12 patients on adjuvant chemotherapy for early breast cancer and 20 HCPs | eRAPID (Web-based) | Weekly | ePROM self-reporting ( CTCAE), feedback to patients with self-management advice, feedback to HCPs before clinic with email alerts, and EMR integration | Field testing | Key themes ( patient perspectives): eRAPID system increased knowledge and confidence, providing a ‘safety net’; and supported decision-making and coping strategies. |
| 7 | Taylor et al [38] (2017) | 15 HCP | PainCheck (Web-based) | - | Electronic pain monitoring from home, and real-time feedback to HCPs | Integrating electronic pain monitoring into palliative care service – HCPs needs | HCPs were uncertain about integration since they were unsure of their utility, and how to interpret and respond. HCPs found it difficult to interpret without patients’ full clinical history. HCPs’ beliefs in existing practice may affect how they view the integration of electronic pain monitoring. |
| 8 | Hansen et al [70] (2020) | 16 patients with haematological cancers on follow-up (participating in an RCT) | ePROM (web-based) | 1 week before clinic encounters | ePROM self-reporting (EORTC QLQ and outcomes and experience questionnaire), available to HCPs in the clinic, and integrated into EMR | Patient experience | Patients felt alienated if ePROM did not resonate with their experience. Patients reported that HCPs rarely used ePRO reports. |
| 9 | Cox et al [36] (2011) | 13 HCPs | HealthHUB | Daily (ESAS) and weekly (EQ-5D) | ePROM self-reports (ESAS and EQ-5D), real-time feedback with alert to HCPs, and feedback to patients with self-management advice | Acceptability | HCPs felt ePROM provided a better record of patients’ symptom experience, empowered patients to report symptom experience, and was inappropriate for older and deteriorating patients. HCPs were concerned it may reduce the opportunity for a face-to-face assessment. |
| Mixed method studies | | | | | | | |
| 10 | Tolstrup et al [50] (2020) | 57 patients with melanoma receiving immunotherapy (participating in an RCT) | AmbuFlex (tablet-based) | Weekly | ePROM self-reporting (PRO-CTCAE),  real-time feedback to HCPs but viewed only at clinic encounters, and patient feedback on when to contact HCPs | Patient and HCP experiences | Patient perceptions: AmbuFlex increased awareness of symptoms, felt more in control and improved communication with HCPs.  HCP perceptions: more time-consuming, preferred EMR integration, felt it helped detection of problems earlier and useful to set agenda during consultations. |
| 11 | Girgis et al [62] (2017) | 35 cancer patients on treatment or follow-up and 5 HCPs | PROMPT-Care | 2-4 weekly (on treatment), monthly (on follow-up | ePROM self-reporting at the clinic and at home (ESAS and SCNS-ST9), HCP feedback – reviewed in the clinic, and tailored self-management resource for patients | Feasibility and acceptability | Patients felt PROMPT-care helped them express themselves and reflect, improving communication and recognizing their problems. HCPs viewed it as a screening tool to prioritize problems to discuss and prepare for the consultation, enabling a deeper understanding of patients’ issues. |
| 12 | Maguire et al [80] (2008) | 35 nurses | ASyMS (mobile phone-based) | Twice daily and ad hoc | ePROM self-reporting (six chemotherapy-related symptoms and temperature), real-time feedback to HCPs, risk-stratified alerts via pager to HCPs, and tailored self-management advice to patients | Nurse perspectives | 43% of nurses perceived that it improved the management of symptoms; 62% considered that it resulted in early detection of symptoms; 50% felt that timely intervention resulted due to alerts. Mixed perception of the impact on workload. |
| 13 | Moradian et al [40] (2018) | 10 patients with any colorectal cancer or lymphoma receiving chemotherapy | ASyMS, (mobile phone-based) | - | ePROM self-reporting, real-time feedback to HCPs, risk-stratified alerts to HCPs, and tailored self-management advice to patients | Usability | 80% (8/10) of participants were highly motivated to use the ASyMS.  Patients indicated it would improve communication with HCP and quicker management of symptoms. |
| 14 | Sundberg et al [65] (2015) | 9 patients with prostate cancer receiving radiotherapy | ICT-platform (mobile phone or tablet) | Daily | ePROM self-reporting, risk-stratified alerts to nurses via text message, and evidence-based self-management advice to patients | Feasibility and acceptability | Patient perspectives: symptom reporting daily created a sense of security for patients; patients appreciated the messaging function to express needs; the alert system created a sense of collaboration with HCP.  HCP perspectives: concern over increased workload and uncertainty over who was responsible for responding to alerts. |
| 15 | Brochmann et al [43] (2016) | 118 patients with myeloproliferative neoplasms (107 completed ePROM) | Web-based | Monthly | ePROM self-reporting (EORTC QLQ-C30, SF-36, BFI, and MPN-SAF), merged with blood test reports, and summary available to HCPs | Feasibility | Patients with higher symptom burden and lower HRQOL reported more frequently.  Patients appreciated free text to communicate symptoms. Time and calmness at home enabled reflection. |
| 16 | Biran et al [59] (2020) | 9 patients with relapsed and refractory multiple myeloma on active treatment | Medocity Home Health App (mobile app) | Weekly and ad hoc | ePROM self-reporting (PRO-CTCAE), real-time feedback and alerts to HCPs, and self-management advice for patients | Acceptability and appropriateness | Patients felt ePROMs helped to reflect and increase awareness of their symptoms. HCPs and patients felt longitudinal data (graphs) helped. HCPs required more specific alerts. HCP theorized benefits: ePROMs improved treatment adherence, empowered patients to communicate, improved relationship with HCP, and gave patients more control through symptom awareness. |
| 17 | Crafoord et al [49] (2020) | 149 patients with breast (BC) cancer on neoadjuvant chemotherapy and patients with prostate cancer (PC) on radiotherapy | Interaktor App | Daily on weekdays up to 18 weeks | ePROM self-reporting (based on MSAS), real-time feedback and risk-stratified SMS alerts to HCPs, self-management advice for patients, and graph with time trends for patients and HCPs | Usage and user experiences | 96% of BC and 75% of PC used free text. Some patients reported it was a negative reminder of their illness, especially when they felt well.  Patients felt monitored, cared for, safe, and had continuous contact with HCP. Daily reporting supported reflecting on their well-being. Graphs helped patients to monitor their well-being. |
| 18 | Zivanovic et al [41] (2020) | 102 cancer patients undergoing ambulatory minimally invasive surgery – gynaecology service | Automated phone calls or online platform | Daily, post-op days 2 to 6 | ePROM self-reports (PRO-CTCAE) and feedback alerts to HCPs | Feasibility and response rate,  qualitative feedback interviews | Responder rate - 67%. 84% of patients did not want to be contacted for moderate symptoms.  Patients felt they were not forgotten and comforted that they were monitored. Patients felt that it removed the burden of deciding when to contact HCP. |
| 19 | Whitehead et al [51] (2020) | 24 patients with breast or colorectal on chemotherapy after surgery | SAM  (web app) | Daily | ePROM self-reporting (8 symptoms + temperature), self-management advice for patients, graph and time trend for patient and HCPs, and risk-stratified alerts to HCPs | Patient experience | Average daily completion over 3 cycles of chemotherapy - 59%.  Patient perceptions: increased awareness of symptoms, felt secure and reassured due to alert facility and supporting symptom management. |
| 20 | Gustavell et al [52] (2020) | 26 patients undergoing pancreaticoduodenectomy surgery for cancer | Interaktor app | Daily for 4 weeks and up to 6 months | ePROM self-reporting (12 symptoms related to surgery + 3 for chemotherapy), real-time feedback and risk-stratified text message alerts to HCPs, self-management advice for patients, and graph with time trends for patients and HCPs | Adherence and patient experience | Mean adherence was 82.2%. All patients triggered alerts, and most used free text messaging. Thematic analysis identified “being seen as a person” as the overarching theme, and “getting your voice heard”, “having access to an extended arm of health care,” and “learning about own health” as subthemes. |
| 21 | Dawes et al [53] (2015) | 20 patients undergoing colorectal surgery (majority for cancers) | ePROM (tablet-based) | Daily for 7-14 days after discharge | ePROM self-reports (8 symptom domains) and real-time feedback to HCPs | Feasibility of real-time symptom surveillance | 63% compliance with reporting. Themes: awareness of the recovery process, activation and participation in post-operative recovery, shared goals and understanding recovery period, sense of connection to the surgical team, and improved patient experience. |
| 22 | Snyder et al [55] (2013) | 47 patients with breast or prostate cancer on treatment and 11 HCPs | PaitentViewpoint, (web-based) | Every 2 weeks | ePROM self-reporting (PROMIS, EORTC BR23, and EPIC), feedback to HCPs (viewed before clinic visit), and integrated into EMR | Usability testing – patients and HCPs | Patients’ perception of usability was rated higher when HCPs viewed reports. Patients used self-reports to express and communicate their concerns. HCPs used reports to identify concerns and perceived it improved the quality of care. HCPs viewed reports from 51% of patients. HCPs preferred graphic scores with guidelines to interpret the reports and viewing in EMR. |
| 23 | Maguire et al [66] (2020) | 18 patients with malignant mesothelioma and 11 HCPs. | ASyMSmeso (ASyMS for patients with malignant mesothelioma) (mobile app) | Daily and ad hoc | ePROM self-reporting, real-time feedback to HCPs, risk-stratified alerts to HCPs, and tailored feedback with self-management advice for patients | Feasibility and acceptability. PROMs (LCSS-Meso, SPARC, TAM) at baseline, 6 weeks and at the end | Compliance rate - 88.5%. Improvement in psychological need on SPARC scale and usefulness domain of TAM.  Themes: patients felt reassured due to a sense of connectivity. HCPs felt the system helped prioritize care, the alerting system enabled early management and change in management approach to anticipatory and preventative. |
| 24 | Lee et al [57] (2022) | 580 patients receiving chemotherapy or radiation therapy | ePROM app | Every week for 3 weeks | ePROM self-reporting (PRO-CTCAE) and summary report feedback to patients with graphs and information on self-management | Factors associated with adoption and compliance | 27.4% adopted with good compliance and 44.5% with poor compliance.  Adoption was associated with:   1. Ease of app use 2. Ease of symptom input 3. “Helpful to recognize my health” 4. Helpful to obtain information to manage symptoms 5. Useful to notify clinicians of symptoms   Good compliance was associated with “helpful to recognize my health” and “manage symptoms during treatment.” All patients agreed that feedback from clinicians would reinforce their compliance. Poor compliance group reported reporting fatigue and ePROMs poorly reflecting their health status. |
| Descriptive studies | | | | | | | |
| 25 | Daly et al [74] (2022) | 217 patients with solid tumor or lymphoma starting chemotherapy | InSight Care (web-based) | Daily | ePROM self-reporting (PRO-CTCAE) and risk-stratified alerts to HCPs | Alert prevalence, preference for ePROM reporting, symptom data capture with daily vs weekly monitoring | 45.1% of red alerts were not preceded by yellow in the previous 7 days.  Three-fold higher probability of an acute event within 7 days after a red alert was triggered.  A higher number of red alerts was associated with a higher likelihood of acute events. |
| 26 | Judson et al [77] (2013) | 286 patients with lung, gynecologic, breast, or genitourinary cancer starting a new chemotherapy | STAR (web-based) | Weekly, anytime between and at clinic encounters | ePROM self-reporting (CTCAE, EQ-5D, and ECOG PS), real-time alerts sent to nurses, and feedback to HCPs at clinic encounters | Longitudinal compliance rates | Average compliance - monthly 83%, weekly 62%.  Compliance rate higher in first 16 weeks. Lung and genitourinary cancer patients showed better compliance than breast cancer patients. Baseline ECOG PS did not affect compliance. Patients with later-stage cancers showed a higher likelihood of compliance (OR 1.19, P = 0.001). |
| 27 | Innominato et al [73] [2021) | 31 patients receiving chemotherapy for advanced cancer | Web-based eHealth platform | Daily | ePROM self-reporting (19-item MDASI) | Daily sampling compared with alternate days (q2d), every third day (q3d), or once a week (q1w) sampling in terms of average symptom intensity and identification of severe symptoms (initial 42 days on study) | “Nausea,” “vomiting,” and “interference with mood” showed larger variations for different sampling intervals. Compared to daily sampling, q2d, q3d, or q1w sampling resulted in MDASI difference > 1 being observed in 0.76%, 2.72%, and 11.93% patients, respectively. Symptom severity (≥ 7): compared with daily sampling, 14.6%, 27.8%, and 55.6% of severe symptoms were missed in q2d, q3d and q1w sampling, respectively. |
| 28 | Andikyan et al [56] (2012) | 49 patients who underwent laparotomy for gynecologic cancers | STAR (web-based) | Weekly for 6 weeks | ePROM self-reporting (CTCAE and EORTC QLQ-C30), real-time feedback email alerts to HCPs, and feedback to HCPs at postoperative clinic encounters | Pilot feasibility study | 82% completed 4/7 sessions of STAR. Alerts were generated for 51% of patients with 25 telephone contacts. 26 patients completed the survey: 64% agreed that STAR made it easier to remember symptoms, 63% improved discussion, 46% improved communication, and 46% felt more in control of their care. |
| 29 | Wintner et al [48] (2015) | Patient with cancer receiving chemotherapy.  113 patients used clinic-ePROM and 45 home-ePROM | CHES (tablet-based) | Weekly | ePROM self-reporting (EORTC QLQ C-30) and HCPs could access cross-sectional or longitudinal reports. | Feasibility and user-friendliness. | 82.2% of home users wanted a discussion of results with HCPs. 77.8% of home users wanted to view their results. 57.8% of home users felt cared for at home. 44% agreed that it was advantageous to have ePROMs always available to report. 91.1% of home users considered ePROMs a useful tool to inform HCPs of their health status, compared with 64.6% of clinic users. |
| 30 | Basch et al [54] (2005) | 80 patients with gynecologic malignancy starting chemotherapy | STAR (web-based) | In the clinic or anytime from home for 8 weeks | ePROM self-reporting (CTCAE, ECOG PS, and EQ-5D), feedback to  HCPs with e-mail alerts, reports available at clinic encounters, and feedback advice for patients to contact HCPs | The pattern of use,  patient  and clinician feedback. | 83% logged in to STAR from home.  90% of patients self-monitored report.  86% entered free-text diary information.  Better ECOG PS was associated with a greater number of logins.  Home users triggered 42/57 of alerts between visits.  Most patients felt it improved communication (85%), improved discussion with HCPs (90%), helped them remember symptoms (94%), and felt more in control (77%). |
| 31 | Basch et al [75] (2007) | 107 patients with thoracic malignancy starting chemotherapy | STAR (web-based) | In the clinic or anytime from home | ePROM self-reporting (CTCAE, EQ-5D, and KPS), feedback to HCPs with e-mail alert, report available at clinic encounters, and feedback advice for patients to contact HCPs | Feasibility, patient satisfaction, and nursing survey | Mean adherence was 78% (during clinic visits); 15% actively reported from home.  Prior computer experience was significantly associated with adherence. Patient perceptions: 84% agreed it was easier to remember symptoms at clinic visits, 77% improved discussion with HCPs, 51% improved communication with HCPs, and 60% felt more in control. |
| 32 | Basch et al [63] (2020) | 495 patients with any advanced or metastatic cancer receiving chemotherapy, targeted oral therapy, and/or immunotherapy (participating in an RCT) | PRO-TECT (web-based or IVR) | Weekly for 1 year | ePROM self-reporting (PRO-CTCAE and ECOG PS), self-management education for patients, e-mail alerts to nurses, and feedback reports available for HCPs at the clinic | User experience survey at 3 months and at the completion of the study. HCP feedback after 6 months. | 77% of patients felt the process improved discussions with their care team; 80.7% stated that their doctor or nurse used the symptom information; 84% noted that it made them feel more in control of their care. The ePROMs completion rate was associated with higher perceived comprehension, general usability, meaningfulness, actionability, clinical utility, and self-efficacy. Prior internet use and higher education negatively correlated with meaningfulness or relevance and communication or actionability. |
| 33 | Denis et al [39] (2014) | 42 patients with lung cancer after treatment without disease progression | Web application | Weekly | ePROM self-report (ten symptoms and weight loss), email alerts to HCPs, and HCP feedback with graphical display | Pilot feasibility study | Mean monthly compliance - 94 %; mean weekly compliance - 79 %.  Patients were reassured, knowing that their oncologist followed the ePROM reports.  Of 43 alerts, 22 were due to additional comments sent by email to HCPs. |
| 34 | Coolbrandt et al [76] (2021) | Patients with gastrointestinal or breast cancers starting first systemic therapy and  22 HCPs | Mynexuzhealth application (web-based) | - | ePROM self-reports (treatment-related symptom), feedback alerts to HCPs, self-management advice for patients with advice on when to contract HCPs, integrated into EMR | Clinical relevance of real-time monitoring of alerts | 44.4% of patients had no intention to contact HCP despite the automated feedback. Nurses rated the clinical relevance of alerts higher than physicians. The highest agreement by HCPs was for “real-time monitoring improved quality of life,” “ without real-time alerts, symptoms would have a negative impact on quality of life,” and “real-time feedback promotes the favorable course of treatment.” |
| 35 | Falchook et al [45] (2016) | 22 patients with head and neck cancer receiving curative radiation therapy | Mobile app | Daily | ePROM self-report (symptoms from 5 domains) and feedback to HCPs reviewed weekly at clinic encounters | Feasibility study | Median reporting compliance -71%.  56% of symptom reports were submitted during nonclinical hours. Symptom reporting was at least 3 times more often than weekly reporting. |
| 36 | Bae et al [47] (2018) | 101 patients on chemotherapy | PRO-SMART (mobile phone-based) | Daily monitoring during 2 cycles of chemotherapy | ePROM self-reporting (AEs, diet, and exercise) and feedback to HCPs with graphs viewed at clinic encounters | Feasibility and accessibility | Accessibility during 2 cycles of chemotherapy - 90.1%.  The number and grade of AEs and the numerical scale of pain recorded in EMR increased after PRO-SMART compared with usual care.  More patients felt it enabled them to notify HCPs of symptoms and thoroughness of management but was not statistically significant. |
| 37 | Rasschaert et al [64] (2021) | 168 patients with solid tumors of any stage and on systemic antineoplastic treatment | AMTRA (web-based with a mobile app) | Daily or ad hoc | ePROM self-reporting (PRO-CTCAE), tailored self-management information for patients, real-time feedback to HCPs with graphs and email alerts, and integrated into EMR | Feasibility and accessibility | Five toxicities (nausea, constipation, loss of appetite, fatigue, and dyspnoea) showed a reduction in grade over time. 64% of patients generated alerts. 65% of patients completed the survey after 3 months: 79% felt it improved communication with HCP, and 66% felt it helped gain therapeutic insights. |
| Quasi-experimental studies | | | | | | | |
| 38 | Girgis et al [8] (2020) | 328 patients with solid tumors receiving active treatment or follow-up care  1312 (propensity score matched) controls | PROMPT-Care (web-based) | Monthly | Intervention - ePROM self-reporting (distress thermometer, ESAS, and SCNS-ST9), real-time feedback to HCPs with email alerts (summary, longitudinal data, recommended action, and referrals), self-management resource for patients, and integrated into EMR  Control - usual care | Primary - ED presentations  Secondary - total time on chemotherapy and referral to allied health services | ED visits were significantly reduced by 33% in the PROMT-Care group.  Time on chemotherapy did not differ between the intervention and control groups.  Allied health referrals were also not significantly different.  32% of feedback reports reviewed vs 44% when provided with clinical alerts. Nurse vs oncologist review of feedback reports: 82% vs 17%, respectively. |
| 39 | Simon et al [82] (2021) | 7165 patients (4195 pre-implementation and 2970 post-implementation) undergoing cancer surgery | Recovery Tracker (RT) | Daily for 10 post-op days | ePROM self-reporting (CTCAE), feedback to patients to contact HCPs, and risk-stratified alerts to the surgical team | Urgent care centre (UCC) visits with and without hospital admission within 30 days of surgery | 67% submitted at least 1 RT survey, and 48% submitted at least 4. 49% triggered at least 1 alert. Risk reduction in the post-RT period for UCC visits without readmission - 22% (OR 0.78, P = .047). 1991 patients were considered responders (completing at least one survey). Risk reduction in responders - 42% (OR 0.58, P = 0.007), number needed to treat = 56. Increase in nursing calls post-RT – 34% |
| 40 | Hough et al [91] (2021) | 387 Patients receiving chemotherapy and NK-1 receptor antagonist as antiemetic.  894 historic controls | Chemotherapy remote care monitoring program (CRCMP) | Daily for 7 days | Intervention - ePROM self-reporting (based on MASCC antiemesis tool), and feedback alerts to the clinical pharmacist | Unplanned health utilization for nausea or vomiting: urgent care, ED, inpatient care, or observation area within 14 days | The response rate was 94%. Overall unplanned utilization rate (encounters per dose of NK-1 antagonist) was lower in CRCMP compared with historic control: 6.68% vs 4.53% (P = 0.02). Urgent care visits were lower in CRCMP: 3.17% vs 0.93% (P = 0.0003). Admission for nausea was lower in CRCMP: 0.63% vs 0.35% (P = 0.33). |
| Randomised control studies | | | | | | | |
| 41 | Absolom et al [78] (2021) | 508 colorectal, breast or gynaecological cancers patients starting chemotherapy | eRAPID (Web-based) | Weekly and ad hoc for 18 weeks | Intervention - ePROM self-reporting, self-management advice for patients with prompts to contact HCPs, real-time feedback to HCPs with email alerts, and integrated into EMR  Control – usual care | Primary - FACT-PWB at 6, 12 and 18 weeks.  Secondary – self-efficacy, HRQOL, process outcomes, and cost-effectiveness | There was a significant positive effect of eRAPID at 6 and 12 weeks but not at 18 weeks on FACT-PWB.  For early-stage disease, there was a positive effect for eRAPID at 6, 12 and 18 weeks. There was a significant increase in self-efficacy in the eRAPID arm at 18 weeks.  HRQOL on EQ-VAS was better in the eRAPID arm at 12 and 18 weeks; EORTC QLQ-C30 scores showed better symptom control at 12 weeks in the eRAPID arm.  No difference in process outcomes (chemotherapy delivery, hospital admissions, and acute oncology assessments) between groups.  HCPs reviewed 81.4% of reports. Patient adherence was associated with HCP use of eRAPID. |
| 42 | Basch et al [4, 5] (2016, 2017) | 766 cancer patients starting chemotherapy (227 computer-inexperienced and 539 computer-experienced participants) | STAR (web-based) | Weekly | Intervention - ePROM self-reporting (CTCAE and EQ-5D), feedback to  HCPs with automated e-mail alerts, reports available at clinic encounters, and feedback advice for patients to contact HCPs.  Control – usual care | Primary - EQ-5D HRQOL at 6 months,  Secondary - survival at 1 year, time to first ER visit, and time receiving active cancer treatment | HRQOL scores improvement (any improvement) - 34% v 18% (P = 0.001) favouring STAR.  Improvement in HRQOL (> 6 points) - 21% v 11% (P = 0.001) favouring STAR.  Fewer participants visited ER in the STAR arm than usual care: 34% v 41% (significant only in computer inexperienced).  Fewer hospitalised in STAR arm: 45% vs 49% (significant only in computer inexperienced)  Median overall survival: 31.2 months in the STAR group vs 26.0 months (P = 0.03), favouring the STAR group. |
| 43 | Mooney et al [81] (2014) | 250 cancer patients starting a new chemotherapy | Automated phone-based symptom reporting (IVR) | Daily for an average of 45 days | Intervention – ePROM self-reporting (10 symptoms), email alerts to HCPs, and HCP feedback reports with graphs  Control - completed automated reporting but no alerts or feedback reports to HCP | Symptom severity and distress, provider-initiated contact, and provider-initiated changes | Daily call adherence was 65.0 %.  No significant difference between treatment and control groups for unscheduled contacts or provider-initiated contacts. No difference in symptom severity or distress scores.  Despite receiving a high number of alerts, few follow-up contacts were initiated by HCPs. |
| 44 | Mooney et al [85] (2017) | 358 patients with cancer starting chemotherapy | IVR | Daily | Intervention - ePROM self-reporting, email alerts to HCPs with decision support for nurses, and tailored self-  management advice for patients  Control – automated symptom reporting without self-management advice or alerts to HCPs | Primary - overall symptom severity  Secondary -individual symptom severity | Significant decrease in symptom severity across all symptoms in the intervention group compared with the control group. Significant reduction in severe and moderate symptoms days in the intervention group. Patients contacted HCPs only 5% of the times recommended. |
| 45 | Egbring et al [69] (2016) | 139 patients with early breast cancer receiving adjuvant chemotherapy | Mobile app | Daily for 6 weeks | Arm A - control group with usual physician support  Arm B - ePROM self-reporting (PRO- CTCAE and ECOG PS)  Arm C – ePROM self-reporting + physician support | Daily functional activity and symptoms over 6 weeks | Daily functional activity stabilised only in arm C and decreased in other arms. Arm C reported a greater number of distinct symptoms in the app than in the paper questionnaire completed at each clinic visit compared with the other two groups. |
| 46 | Yount et al [86] (2014) | 253 lung cancer patients (Stage III or IV) receiving chemotherapy | IVR | Weekly for 12 weeks | Intervention – ePROM self-reporting (FACT-FLSI) and automated e-mail alerts to HCPs with longitudinal graphical display of reports at clinic visits  Control - ePROM reporting only | Primary - SDS and FACT-FLSI  Secondary - FACT-G, FACIT-TS-PS, self-efficacy, and medical care utilisation | No significant difference between groups in mean SDS.  The intervention group had significantly lower scores on comprehensive care and decision-making (FACIT-TS-PS).  The intervention group made more phone calls to HCPs.  A high number of alerts in the intervention group (>1323 in 12 weeks). |
| 47 | Graetz et al [46] (2018) | 48 patients receiving aromatase inhibitors (AI) as adjuvant treatment for breast cancer | PCM (Tablet-based app) | Weekly | Group 1: PCM + weekly reminders  Group 2: PCM without reminders  PCM – ePROM self-reporting (FACT-ES and MARS-1 scale), email alerts sent to HCPs, and integrated into EMR | Primary - AI adherence at 6-8 weeks (MMAS-4)  Secondary - FACT-ES | Group 1 had a higher proportion of weekly logins than group 2 - 73.5 vs. 37.6% (P < 0.05).  Group 1 had higher weekly alerts than group 2 – 2.4 vs 1.7(P < 0.05). 95% of alerts were responded to within 48hrs.  Group 1 showed higher adherence to AI than group 2 – 100% vs 72.7%(P < 0.05). There was no difference in symptom burden. |
| 48 | Graetz et al [44] (2018) | 29 patients with ovarian, fallopian or primary peritoneal cancer scheduled for open surgical staging | PCM (Tablet-based) | Ad hoc | Group 1: PCM + reminders (daily to weekly)  Group 2: PCM without reminders  PCM - ePROM self-reporting (12 symptoms), real-time feedback with alerts (text or email) to HCPs, and integrated into EMR. | HRQOL (SF-12) at baseline and 30 days after discharge | Group 1 used the PCM app more times than group 2 in all weeks - 46% vs 15% (P = 0.05) in week 2.  In group 1, the mean mental health composite score increased (P = 0.08), and the mean physical health decreased (P = 0.003) but remained stable in group 2. |
| 49 | Spoelstra et al [84] (2013) | 119 patients with a solid tumour on nonhormonal oral chemotherapy treatment | AVR (phone-based) | Weekly for 10 weeks | Group 1 - AVR + symptom management tool kit (SMT)  Group 2 - AVR + SMT + nurse feedback for symptoms management and adherence  Group 3 - AVR + SMT + nurse feedback only for adherence | Symptom Experience Inventory and medication adherence | Within group pairwise comparison between baseline and 10 weeks scores: group 1- a decrease of 4.74(P = 0.03), group 2 - a decrease of 6.76 (P = 0.03) and group 3 - a decrease of 2.16 (P = 0.39). AVR alone was as effective for symptom management and adherence as AVR with nurse intervention. The baseline symptom severity was significantly different between groups. |
| 50 | Cleeland et al [83] (2011) | 100 patients undergoing thoracic surgery for primary or secondary cancers | IVR | Twice a week after discharge for 4 weeks | Intervention - ePROM self-reports (MDASI) and email alerts sent to nurses  Control - IVR without alerts to nurses | Symptom threshold events,  mean symptom severity | 33 patients in the intervention group generated an alert vs 36 patients (who would have generated alerts) in the control group.  84% of alerts were reviewed and 60% responded to by phone consultations.  There was a significant reduction in symptom threshold events -19% in the intervention group vs 8% in the control group. Symptom severity was not different between the groups. |
| 51 | Shiroiwa et al [71] (2022) | 102 patients with metastatic cancer on chemotherapy or immunotherapy | ePROM (tablet-based) | Paper PROM -baseline and the day of 2 chemotherapy cycles  ePROM - baseline, days 3, 7,10, 14, and day of chemotherapy | Intervention - ePROM (EQ-5D-5L and EORTC QLQ-C 30)  Control - paper PROM (EQ-5D-5L and EORTC QLQ-C 30) | Primary - EQ-5D-5L  Secondary - EORTC QLQ-C 30 | HRQOL at the same time points were not statistically different between the ePROM and paper-PROM groups. Quality-adjusted lifedays (AUC of longitudinal scores) for EO-5D-5L was lower in the ePROM group - the difference between the groups was 1.36 (P = 0.0021). AUC for EORTC QLQ-C 30 was not significantly different between the groups. |
| 52 | Tolstrup et al [68] (2022) | 146 patients with metastatic melanoma on checkpoint inhibitors | AmbuFlex, (tablet-based) | Weekly for 24 weeks | Intervention – ePROM self-reporting (PRO-CTCAE), real-time feedback to HCP but viewed only at clinic encounters, and feedback to patients to contact HCPs  Control – usual care. | EQ-5D-5L and FACT-M at baseline, 24, and 48 weeks | The intervention group made more phone calls and more unplanned visits. The mean EQ-5D score in the intervention group was significantly higher than the control at 48 weeks (P = 0.05) but not at 24 weeks.  FACT-M scores were slightly higher in the intervention group compared to the control (P = 0.12). |
| 53 | Mir et al [9] (2022) | 559 patients starting oral anticancer agents | CAPRI application (web-based) | First month -weekly; 2-4 months - alternate weeks; from 5th month, every 3 weeks | Intervention - ePROM self-reporting (symptoms), self-management advice for patients, real-time feedback to nurses with alerts, and clinical decision tools  Control - usual care | Primary - RDI  Secondary - MEMS, EORTC QLQ-C 30, survival, CTCAE, and PACIC | RDI mean – 93.4% in the CAPRI arm vs 89.4% in the control arm (P = 0.043).  Grade ≥ 3 toxicities: 27.6% in the CAPRI arm vs 36.6% in the control arm (P = 0.02).  Hospitalisation: 22.8% in the CAPRI arm vs 31.7% in the control arm (P = 0.02).  ED visits: 15.1% CAPRI arm vs 22.0% in the control arm (P = 0.04). |
| 54 | Maguire et al [79] (2021) | 829 patients with Hodgkin or non-Hodgkin lymphoma, breast or colorectal cancers on adjuvant chemotherapy | ASyMS (mobile app) | Daily and ad hoc up to six cycles of chemotherapy | Intervention - ePROM self-reporting (10 symptoms), real-time feedback with risk-stratified alerts to HCPs, and tailored feedback with self-management advice to patients  Control - usual care | Primary - MSAS  Secondary - SCNS-SF34, FACT-G, CASE-Cancer, WLQ, and STAI-R | Lower MSAS score favouring intervention group (Cohen’s effect size 0.5). FACT-G scores were higher, favoring the intervention group (mean difference 4.06, P < 0.001). Significantly better STAI-R trait anxiety scores, lower supportive care needs and greater self-efficacy in the intervention group. |
| 55 | Pappot et al [87] (2021) | 682 patients with breast cancer on adjuvant chemotherapy (cluster randomized trial) | AmbuFlex | Before each chemotherapy for 6 cycles | Intervention - ePROM reporting (PRO-CTCAE), and real-time feedback to HCPs  Control - standard care | Primary - chemotherapy adjustments  Secondary -hospitalization and febrile neutropenia | No difference in chemotherapy adjustments between the arms. No difference in hospitalization or febrile neutropenia between the arms. |
| 56 | Basch et al [7] (2022) | 1191 patients with any metastatic cancer receiving chemotherapy, targeted oral therapy, and/or immunotherapy | PRO-TECT (web or telephone-based) | Weekly for 1 year | Intervention - ePROM self-reporting (PRO-CTCAE, ECOG PS, oral intake, and financial challenges), self-management advice for patients, and HCP feedback with time trends and e-mail alert  Control - usual care | Primary - overall survival  Secondary - physical function at 3 months, symptom control, and EORTC QLQ-C30 | At 3 months, the mean change from baseline (EORTC QLQ-C30) for physical function (mean difference 2.47, P = 0.02), symptom control (mean difference 2.56 P = 0.002), and HRQOL (mean difference 2.43, P = 0.002) were better in the intervention group; the difference remained significant for up to 9 months.  91.5% completed weekly reports.  Nurses responded immediately to 59.1% of alerts. 76.8% of patients felt more in control of their care, and 72.4% felt it improved discussion with HCPs. |
| 57 | Zhang et al [89] (2022) | 278 patients receiving immunotherapy | ePROM app | Weekly | Intervention - ePROM self-reporting (CTCAE), self-management advice for patients, and HCP feedback alerts via email and text message  Control - usual care | Incidence and severity of AEs, ED visits, rate of treatment discontinuation EORCT QLQ-C 30, and deaths due to AE | The intervention group had fewer severe AEs (20.6% vs 33.6%, P = 0.01), fewer ED visits (16.3% vs 29.9%, P = 0.01), and fewer treatment discontinuation due to AEs (3.6% vs 11%, P = 0.02). The HRQOL means scores were higher at 6 months for the physical and emotional domains in the intervention group. |
| 58 | Kearney et al [72] (2009) | 112 patients with breast, lung or colorectal cancer starting a new chemotherapy | ASyMS | Twice daily and ad hoc days 1-14 of each cycle | Intervention - ePROM self-reporting (CTCAE), tailored self-management advice for patients, and risk-stratified feedback alerts via pager to HCPs  Control - usual care | Incidence and severity of chemotherapy-related symptoms before cycles 2-5 | Higher incidence of fatigue (OR 2.29, P = 0.040) and a lower incidence of hand-foot syndrome (OR = 0.39, P = 0.031) in the control group compared with the intervention group. |
| 59 | Denis et al [6] (2017) | 121 patients with nonprogressive small cell or non-small cell lung cancer | e-FAP | Weekly | Intervention - ePROM self-reporting (12 symptoms) and HCP feedback with alerts sent by email  Control - standard follow-up | Primary - overall survival (OS)  Secondary - PS at first relapse, PFS, and FACT-L | Median OS: 19 months (intervention group) vs 12 (control group) (P = 0.001). The HRQOL score change from baseline to 6 months was stable or improved in 80.6% of patients in the intervention group compared with 58.6% in the control group. PS at first relapse was 0 or 1 in 75.9% of patients in the intervention group compared with 32.5% in the control group. 86% of patients triggered at least one alert. Unscheduled visits: 58.3% (intervention group) vs 24.6% (control group). Relapses detected between scheduled visits: 72.4% (intervention group) vs 32.5% (control group). |
| 60 | Greer et al [88] (2020) | 181 patients with cancer on oral anti-cancer treatment | Mobile app | Weekly for 12 weeks | Intervention - ePROM self-reporting (adherence and 17 symptoms), physical activity tracking usingFitbit, weekly feedback to clinicians by email, medication reminders, and self-management resources for patients  Control - usual care | Primary - adherence (electronic pill cap), MDASI, and FACT-G  Secondary -MMAS-4, FACIT-TS-PS, ED visits, and hospitalisations | No difference in adherence or other primary outcomes between the two groups.  In patients with baseline adherence problems based on MMAS-4 and with higher HADS-anxiety subscale scores, there was significantly higher mean adherence favoring the mobile app group. Better app engagement was associated with fewer ED visits resulting in hospitalizations. |
| 61 | Fjell et al [90] (2020) | 150 patients with breast cancer undergoing neoadjuvant chemotherapy | Interaktor app | Daily for 18 weeks | Intervention - ePROM self-reporting(14 symptoms), real-time feedback to HCPs with risk-stratified alerts by text messages, and self-management advice to patients  Control - usual care | MSAS and  EORTC QLQ-C30 | In the intervention group, the prevalence of nausea, vomiting and feeling sad was significantly lower at 2 weeks after the end of treatment. The overall symptom distress (MSAS) and physical symptom distress were significantly lower in the intervention group compared with the control group. In EORTC QLQ-C30, nausea, vomiting, appetite loss and constipation symptoms were significantly lower and emotional function was significantly higher in the intervention group compared with the control group. |

AE: adverse event

AUC: area under the curve

AVR: automated voice response phone system

ASyMS: mobile phone, advanced symptom management system

AMTRA: Ambulatory Monitoring of cancer Therapy using an interactive Application

BFI: Brief Fatigue Inventory

CHES: Computer-based Health Evaluation System

CTCAE: Common Terminology Criteria for Adverse Events

CAPRI: Impact of a Monitoring Device for Patients With Cancer Treated Using Oral Therapeutics

CASE: Cancer - Communication and Attitudinal Self-Efficacy scale for cancer

ePROM: electronic patient-reported outcome measure

e-FAP: e-follow-up application

EMR: electronic medical records

EORTC QLQ-C30: European Organisation for Research and Treatment of Cancer Quality-of-life Questionnaire – Core questionnaire 30 items

EORTC BR23: European Organisation for Research and Treatment of Cancer Quality-of-life Questionnaire – Breast-cancer specific module

EPIC: Expanded Prostate Cancer Index short-form

ED: emergency department

ER: emergency room

eRAPID: Electronic patient self-Reporting of Adverse-events: Patient Information and aDvice

ESAS: Edmonton Symptom Assessment Scale

EQ-5D-5L: EuroQol EQ-5D ( 5 domain, 5 level)

EQ-VAS: EuroQoL visual analogue scale

ECOG PS: Eastern Cooperative Oncology Group performance status

FACIT-TS-PS : Functional Assessment of Chronic Illness Therapy– Treatment Satisfaction–Patient Satisfaction

FACT-G: Functional Assessment of Cancer Therapy – General

FACT-B: Functional Assessment of Cancer Therapy – Breast

FACT-C: Functional Assessment of Cancer Therapy – Colorectal

FACT-L: Functional Assessment of Cancer Therapy–Lung

FACT-M: Functional Assessment of Cancer Therapy – Melanoma

FACT-PWB: Functional Assessment of Cancer Therapy-General, Physical Well-Being subscale

FACT-ES: Functional Assessment of Cancer Therapy Endocrine Symptoms

FACT-FLSI: Functional Assessment of Cancer Therapy Lung Cancer Symptom Index

HCP: healthcare provider

HRQOL: health-related quality of life

HADS: Hospital Anxiety and Depression Scale

ICT: Information and communication technology

IVR: telephone-based interactive voice response system

KPS: Karnofsky Performance Status

LCSS-Meso: Lung Cancer Symptom Scare – Mesothelioma

MSAS: Memorial Symptom Assessment Scale

MDASI: MD Anderson symptom inventory

MPN-SAF: myeloproliferative neoplasm symptom assessment form

MARS-1: Medication Adherence Reasons Scale

MMAS-4: Morisky Medication Adherence Scale

MEMS: medication event monitoring system

OR : odds ratio

PCM : Patient Care Monitor

PRO-CTCAE: Patient-Reported Outcomes version of the Common Terminology Criteria for Adverse Events

PROMT-Care: Patient-Reported Outcome Measures for Personalized Treatment and Care

PRO-SMART: Electronic PRO measures using a smartphone

PRO-TECT: Patient Reported Outcomes to Enhance Cancer Treatment

PFS: progression-free survival

PS: performance status

PROMIS : Patient Reported Outcomes Measurement Information System

PACIC: Patient Assessment of Chronic Illness Care

RDI : relative dose intensity (defined as the ratio of the dose actually delivered over time to the prescribed dose intensity)

RCT: randomized controlled trial

RSCL: Rotterdam Symptom Checklist

SAM: Symptom Assessment and Management system

SF-36 : 36-item Short Form survey instrument

SPARC: Sheffield Profile for Assessment and Referral for Care

STAR: Symptom Tracking and Reporting

SCNS-ST9: Supportive Care Needs Survey-Screening Tool 9

SDS: Symptom Distress Scale

SCNS-SF34: Supportive Care Needs Survey Short-Form

STAI-R: State-Trait Anxiety Inventory-Revised

SUPPH: Strategies Used by Patients to Promote Health

SF-12: 12-item Short Form survey instrument

TAM : Technology Acceptance Model measure for eHealth

WLQ : Work Limitations Questionnaire
