# Supplementary material for: Assessing Patient-Reported Outcomes in Routine Cancer Clinical Care Using Electronic Administration and Telehealth Technologies: Realist Synthesis of Potential Mechanisms for Improving Health Outcomes
Source: J Med Internet Res. 2023 Nov 28;25:e48483. doi: 10.2196/48483 (PMC10716761; doi:10.2196/48483)
Supplement: Multimedia Appendix 4 [file jmir_v25i1e48483_app4.docx]

**Quality appraisal using Mixed Methods Appraisal Tool**

1. Qualitative studies (n = 6)

| No. | Methodological quality criteria | Hansen 2020 [70] | Richards 2021 [58] | Warrington 2019 [60] | Snyder 2009 [42] | Duman-Lubberding 2015 [67] | Wu 2016 [61] |
| --- | --- | --- | --- | --- | --- | --- | --- |
| 1.1 | Is the qualitative approach appropriate to answer the research question? | Y | Y^a^ | Y | Y | Y | Y |
| 1.2 | Are the qualitative data collection methods adequate to address the research question? | Y | Y | Y | Y | Y | Y |
| 1.3 | Are the findings adequately derived from the data? | Y | Y | Y | Y | Y | CT^b^ |
| 1.4 | Is the interpretation of results sufficiently substantiated by data? | Y | Y | Y | Y | Y | N^c^ |
| 1.5 | Is there coherence between qualitative data sources, collection, analysis and interpretation? | Y | Y | Y | Y | Y | CT |

^a^Y: Yes

^b^CT: Can’t tell

^c^N: No

1. Quantitative randomised controlled trials (n = 21)

| No. | Methodological quality criteria | Kearney 2009 [72] | Basch 2022 [7] | Basch 2016 [4] | Absolom 2021 [78] | Denis 2017 [6] | Fjell 2020 [90] | Cleeland 2011 [83] | Egbring 2016 [69] | Graetz 2018 [44] | Graetz 2018 [46] | Mooney 2014 [81] | Mooney 2017 [85] | Spoelstra 2013 [84] | Shiroiwa 2022 [71] | Maguire 2021 [79] | Yount 2014 [86] | Greer 2020 [88] | Pappot 2021 [87] | Tolstrup 2022 [68] | Zhang 2022 [89] | Mir 2022 [9] |
| --- | --- | --- | --- | --- | --- | --- | --- | --- | --- | --- | --- | --- | --- | --- | --- | --- | --- | --- | --- | --- | --- | --- |
| 2.1 | Is randomisation appropriately performed? | Y | Y | Y | Y | Y | Y | Y | Y | Y | Y | Y | Y | CT | Y | Y | Y | Y | CT | Y | Y | Y |
| 2.2 | Are the groups comparable at baseline? | CT | CT | Y | Y | N | Y | Y | CT | Y | Y | CT | Y | N | CT | Y | Y | Y | CT | CT | Y | CT |
| 2.3 | Are there complete outcome data? | N | Y | N | N | Y | Y | N | Y | Y | Y | Y | Y | N | Y | N | Y | N | CT | N | Y | Y |
| 2.4 | Are outcome assessors blinded to the intervention provided? | N | N | N | N | N | N | N | Y | N | N | N | N | N | N | Y | N | N | N | N | N | N |
| 2.5 | Did the participants adhere to the assigned intervention? | Y | Y | Y | N | Y | Y | N | Y | Y | Y | Y | Y | Y | Y | Y | Y | N | Y | Y | Y | CT |

1. Quantitative Non-randomised studies (n = 3)

| No. | Methodological quality criteria | Simon 2021 [82] | Hough 2021 [91] | Girgis 2020 [8] |
| --- | --- | --- | --- | --- |
| 3.1 | Are the participants representative of the target population? | Y | Y | Y |
| 3.2 | Are measurements appropriate regarding both the outcome and intervention (or exposure)? | Y | Y | Y |
| 3.3 | Are there complete outcome data? | Y | Y | Y |
| 3.4 | Are the confounders accounted for in the design and analysis? | Y | N | Y |
| 3.5 | During the study period, is the intervention administered (or exposure occurred) as intended? | Y | Y | Y |

1. Quantitative descriptive studies (n = 12)

| No. | Methodological quality criteria | Andikyan 2012 [56] | Basch 2005 [54] | Basch 2007 [75] | Basch 2020 [63] | Bae 2018 [47] | Coolbrandt 2020 [76] | Daly 2022 [74] | Innominato 2021 [73] | Judson 2013 [77] | Falchook 2016 [45] | Rasschaert 2019 [64] | Wintner 2015 [48] |
| --- | --- | --- | --- | --- | --- | --- | --- | --- | --- | --- | --- | --- | --- |
| 4.1 | Is the sampling strategy relevant to address the research question? | CT | Y | Y | Y | Y | Y | Y | Y | Y | Y | Y | Y |
| 4.2 | Is the sample representative of the target population? | N | Y | Y | Y | Y | CT | Y | CT | Y | Y | Y | Y |
| 4.3 | Are the measurements appropriate? | Y | Y | Y | Y | Y | Y | Y | Y | Y | Y | Y | Y |
| 4.4 | Is the risk of nonresponse bias low? | CT | Y | N | Y | Y | CT | CT | CT | Y | Y | N | Y |
| 4.5 | Is the statistical analysis appropriate to answer the research question? | N | Y | Y | Y | Y | Y | Y | Y | Y | Y | Y | ­—^d^ |

^d^—: No statistical analysis

1. Mixed Method studies (n = 13)

| No. | Methodological quality criteria | Biran 2020 [59] | Snyder 2013 [55] | Maguire 2020 [66] | Maguire 2008 [80] | Brochmann 2015 [43] | Crafoord 2020 [49] | Dawes 2015 [53] | Girgis 2017 [62] | Gustavell 2020 [52] | Lee 2022 [57] | Tolstrup 2020 [50] | Sundberg 2015 [65] | Whitehead 2020 [51] |
| --- | --- | --- | --- | --- | --- | --- | --- | --- | --- | --- | --- | --- | --- | --- |
| 1.1 | Is the qualitative approach appropriate to answer the research question? | Y | Y | Y | Y | Y | Y | Y | Y | Y | Y | Y | Y | Y |
| 1.2 | Are the qualitative data collection methods adequate to address the research question? | Y | N | Y | Y | Y | Y | N | Y | Y | Y | Y | Y | Y |
| 1.3 | Are the findings adequately derived from the data? | CT | Y | Y | Y | Y | Y | Y | Y | Y | Y | Y | Y | Y |
| 1.4 | Is the interpretation of results sufficiently substantiated by data? | CT | N | Y | Y | Y | Y | Y | Y | Y | Y | Y | CT | Y |
| 1.5 | Is there coherence between qualitative data sources, collection, analysis and interpretation? | Y | CT | Y | Y | Y | Y | Y | Y | Y | Y | Y | Y | Y |
| 4.1 | Is the sampling strategy relevant to address the research question? | N | Y | Y | Y | Y | Y | Y | Y | Y | Y | Y | N | Y |
| 4.2 | Is the sample representative of the target population? | Y | Y | Y | Y | Y | Y | Y | Y | Y | Y | Y | Y | Y |
| 4.3 | Are the measurements appropriate? | Y | Y | Y | Y | Y | Y | Y | Y | Y | Y | Y | N | Y |
| 4.4 | Is the risk of nonresponse bias low? | Y | Y | Y | Y | Y | Y | Y | Y | Y | Y | Y | Y | Y |
| 4.5 | Is the statistical analysis appropriate to answer the research question? | — | — | Y | — | Y | Y | — | — | — | Y | — | — | Y |
| 5.1 | Is there an adequate rationale for using a mixed methods design to address the research question? | Y | Y | Y | Y | Y | Y | Y | Y | Y | Y | Y | Y | Y |
| 5.2 | Are the different components of the study effectively integrated to answer the research question? | Y | Y | Y | Y | Y | Y | N | Y | Y | Y | Y | Y | Y |
| 5.3 | Are the outputs of the integration of qualitative and quantitative components adequately interpreted? | Y | N | Y | Y | Y | Y | Y | Y | Y | Y | Y | N | Y |
| 5.4 | Are divergences and inconsistencies between quantitative and qualitative results adequately addressed? | ND^e^ | ND | ND | ND | ND | ND | ND | ND | ND | ND | ND | ND | ND |
| 5.5 | Do the different components of the study adhere to the quality criteria of each tradition of the methods involved? | N | N | Y | Y | Y | Y | N | Y | Y | Y | Y | N | Y |

^e^ND: No divergences
